# Sex chromosome aneuploidies give rise to changes in the circular RNA profile: A circular transcriptome-wide study of Turner and Klinefelter syndrome across different tissues

**DOI:** 10.3389/fgene.2022.928874

**Published:** 2022-07-22

**Authors:** Emma B. Johannsen, Jesper Just, Mette H. Viuff, Trine Line Hauge Okholm, Steen B. Pedersen, Katrine Meyer Lauritsen, Christian Trolle, Mette Glavind Bülow Pedersen, Simon Chang, Jens Fedder, Anne Skakkebæk, Claus H. Gravholt

**Affiliations:** ^1^ Department of Molecular Medicine, Aarhus University Hospital, Aarhus, Denmark; ^2^ Departments of Clinical Medicine, Aarhus University, Aarhus, Denmark; ^3^ Otolaryngology-Head and Neck Surgery and Microbiology and Immunology, University of California, San Francisco, San Francisco, CA, United States; ^4^ Steno Diabetes Center, Aarhus University Hospital, Aarhus, Denmark; ^5^ Department of Endocrinology, Aarhus University Hospital, Aarhus, Denmark; ^6^ Centre of Andrology and Fertility Clinic, Department D, Odense University Hospital, Odense, Denmark; ^7^ Research Unit of Human Reproduction, Institute of Clinical Research, University of Southern Denmark, Odense, Denmark; ^8^ Department of Clinical Genetics, Aarhus University Hospital, Aarhus, Denmark

**Keywords:** Turner syndrome, Klinefelter syndrome, genomics, circRNA, lncRNA

## Abstract

**Purpose:** The landscape of circular RNAs (circRNAs), an important class of non-coding RNAs that regulate gene expression, has never been described in human disorders of sex chromosome aneuploidies. We profiled circRNAs in Turner syndrome females (45,X; TS) and Klinefelter syndrome males (47,XXY; KS) to investigate how circRNAs respond to a missing or an extra X chromosome.

**Methods:** Samples of blood, muscle and fat were collected from individuals with TS (*n* = 33) and KS (*n* = 22) and from male (*n* = 16) and female (*n* = 44) controls. CircRNAs were identified using a combination of circRNA identification pipelines (CIRI2, CIRCexplorer2 and circRNA_finder).

**Results:** Differential expression of circRNAs was observed throughout the genome in TS and KS, in all tissues. The host-genes from which several of these circRNAs were derived, were associated with known phenotypic traits. Furthermore, several differentially expressed circRNAs had the potential to capture micro RNAs that targeted protein-coding genes with altered expression in TS and KS.

**Conclusion:** Sex chromosome aneuploidies introduce changes in the circRNA transcriptome, demonstrating that the genomic changes in these syndromes are more complex than hitherto thought. CircRNAs may help explain some of the genomic and phenotypic traits observed in these syndromes.

## Introduction

Turner syndrome (45,X; TS) and Klinefelter syndrome (47,XXY; KS) are human sex chromosome aneuploidies (SCAs). TS lacks the second X chromosome present in females, while KS has an additional X chromosome. Both syndromes present with high variability in phenotype, complicating distinction from females and males of normal karyotypes (Berglund et al., 2019). Many clinical features are common to both syndromes (reviewed in (Gravholt et al., 2019); TS and (Gravholt et al., 2018); KS), which include infertility, hypergonadotropic hypogonadism, altered body composition and weight, and an increased frequency of the metabolic syndrome, type 2 diabetes, osteoporosis and autoimmune diseases. However, other clinical traits are syndrome specific, such as congenital malformations related to the heart, which are prevalent among females with TS.

In 46, XX females and in 47, XXY males, one of the X chromosomes undergoes X chromosome inactivation (XCI) (Disteche, 1995). However, 15% of X-linked genes escape XCI. These so-called “escape genes” are located throughout the X chromosome, but predominate in the pseudoautosomal regions (PAR) of homology between X- and Y-chromosomes (Berletch et al., 2011). The genes residing in these regions have a decreased expression in TS while the opposite is true for KS (Zhang X. et al., 2020). In line with this, the expression of the escape gene *SHOX* correlates with short and tall stature in TS and KS respectively, suggesting an X-dosage dependency (Rao et al., 1997).

Despite an incidence of one in 2,000 females for TS and one in 660 males for KS (Nielsen and Wohlert, 1991; Berglund et al., 2020), the underlying pathophysiology for both syndromes is largely unknown. Recent studies have demonstrated that changes in the transcriptome and methylome may explain part of the biological mechanisms underlying the phenotypes of sex chromosome aneuploidies (SCAs) (Sharma et al., 2015; Raznahan et al., 2018; Zhang X. et al., 2020; Gravholt et al., 2022). Based on fibroblast cell lines, peripheral blood mononuclear cells (PBMCs), amniotic fluid cells and pluripotent human cell lines, an altered expression of genes from both autosomes and sex chromosomes, in part due to global methylation changes, has been suggested in TS (Bakalov et al., 2009; Zhang et al., 2013; Massingham et al., 2014; Rajpathak et al., 2014). Transcriptome alterations in blood, brain and testis tissue, as well as recent studies of the methylome in blood and brain tissue in KS (Wan et al., 2015; Viana et al., 2014; Huang et al., 2015; Zitzmann et al., 2015; Belling et al., 2017; Winge et al., 2018; D'Aurora et al., 2015; Panula et al., 2019; Astro et al., 2021) have further substantiated the belief that SCAs introduce global changes in the methylome and transcriptome. Opposite gene expression patterns, depending on the number of X chromosomes, suggest the existence of genetically modified networks due to X-chromosome dosage (Raznahan et al., 2018; Astro et al., 2021), and as a result, a ripple effect that spreads and affects the genome globally (Zhang X. et al., 2020).

Besides methylation changes and gene expression changes of protein coding genes, several studies have also demonstrated an altered expression of non-coding RNAs, e.g. microRNAs (miRNAs), long non-coding RNAs and small interfering RNAs (siRNAs), in TS and KS (Trolle et al., 2016; Skakkebaek et al., 2018; Zhang X. et al., 2020). Non-coding RNAs have been shown to be important regulators of gene expression and translation by different mechanisms and modulate each other by engaging in competing endogenous RNA (ceRNA) networks (Siniscalchi et al., 2022).

In the realm of non-coding RNAs, circular RNAs (circRNAs) are a newly discovered class of endogenous, single-stranded RNAs. Their 5′ and 3’ termini are covalently closed in a loop structure facilitated by non-linear splicing events termed backsplicing (Salzman et al., 2012). They are abundant in mammalian cells and display developmental stage-, cell type- and tissue-specific expression patterns (Memczak et al., 2013). The exact functionality of many circRNAs remains elusive, but they are known to take part in many, primarily regulatory, processes (Kristensen et al., 2019). The best described functions include binding and sequestering—so-called “sponging”—of miRNAs and interaction with RNA-binding proteins (RBPs), through which they function as protein sponges or inhibitors, and thereby affect both gene expression, translation and protein regulation (Hansen et al., 2013; Memczak et al., 2013). Expressional changes of circRNAs have been observed in several diseases, including cardiovascular disease, diabetes and several cancers (Wang et al., 2016; Stoll et al., 2018; Kristensen et al., 2021). However, circRNAs have, to the best of our knowledge, never been investigated in neither KS, TS nor other SCAs.

**FIGURE 1:**
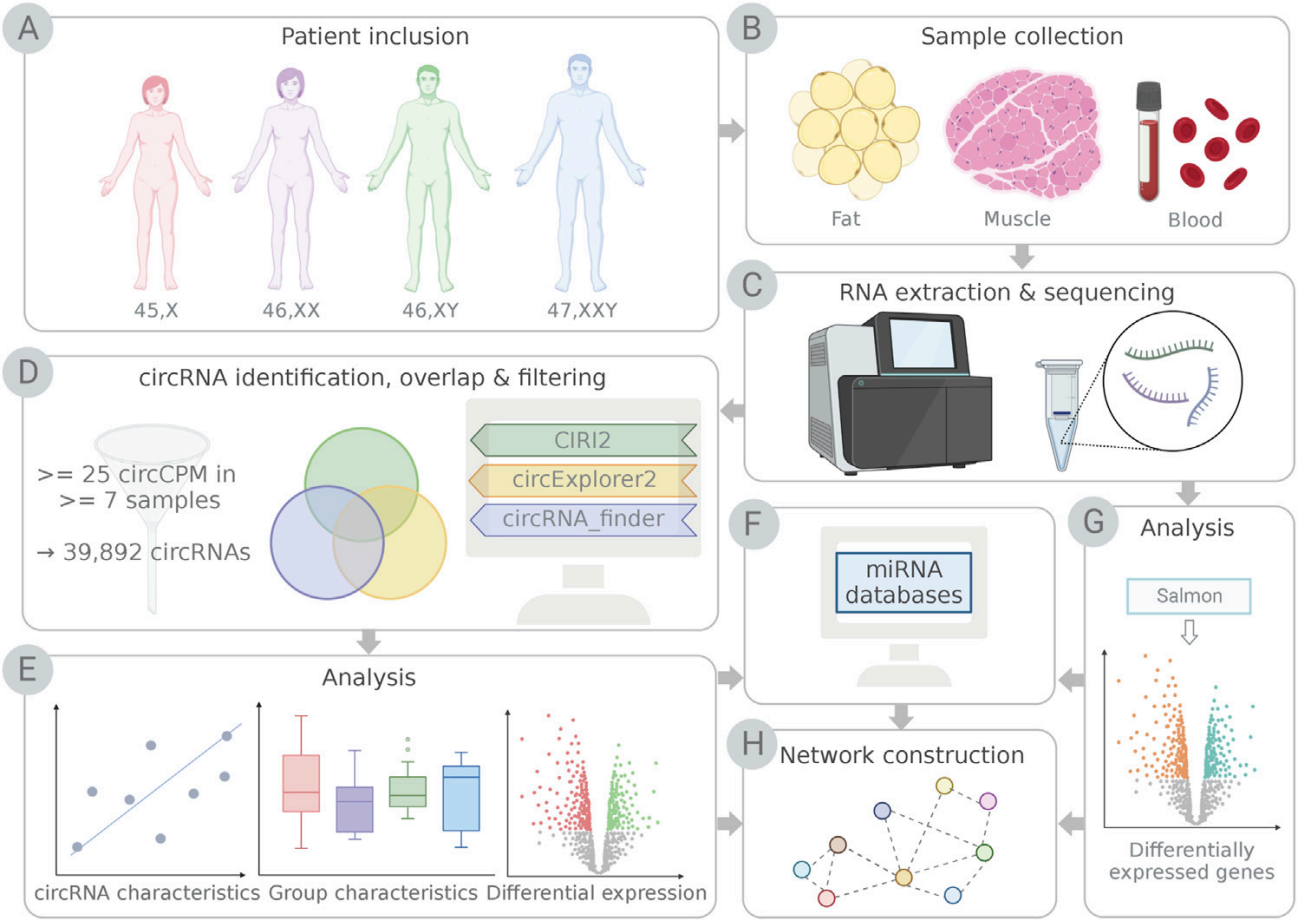
Schematic illustration of the study workflow. **(A)** Participants included females with Turner syndrome (TS; 45,X), female controls (46,XX), male controls (46,XY) and males with Klinefelter syndrome (KS; 47,XXY). **(B)** Samples of fat, muscle and blood were collected, **(C)** RNA was purified and sequenced. **(D)** circRNAs were identified using detection pipelines CIRI2, circExplorer2 and circRNA_finder. Only circRNAs identified by CIRI2 and at least one additional pipeline and expressing ≥25 circCPM in ≥7 samples were kept for further analysis. **(E)** circRNAs were analyzed based on known circRNA characteristics, group characteristics and differential expression. **(F)** Potential micro RNA (miRNA) interactions for differentially expressed circRNAs were identified, and mRNA targets for miRNAs were obtained from miRNA databases and tools. **(G)** Genes from the RNA sequencing data **(F)** identified as targets for miRNAs **(H)** were used for network construction with differentially expressed circRNAs and miRNAs. This illustration was created with BioRender.com.

This study investigates the potential impact of an altered circRNA profile in TS and KS, and the associated clinical traits, by performing comparative analyses of circRNA expression in TS and KS versus female and male controls, respectively, using three tissues of interest: blood, fat and muscle (Figure 1). Identifying possible alterations in the expression profile of circRNAs and other non-coding RNAs in TS and KS will be important to elucidate how these may shape underlying networks that regulate gene and protein levels.

## Materials and methods

### Sample inclusion

Samples included muscle biopsies, fat biopsies and peripheral blood samples from male and female controls, males with Klinefelter syndrome (47,XXY) and females with Turner syndrome (45,X) (Table 1). All cases of TS and KS were verified by karyotyping, and karyotype mosaicism was not included.

**TABLE 1:** Group characteristics.

	*TS (45,X)*	*Female (46,XX)*	*Male (46,XY)*	*KS (47,XXY)*
Number of participants				
Blood	33	34	16	19
Muscle	10	5	15	21
Fat	10	11	15	22
Paired across all tissues*	10	5	14	18
Age	46.1 (31-62)	43.9 (23-68)	38.7 (22-62)	38.8 (21-53)
BMI	28.9 (18.8–47.1)	26.3 (18.9–35.6)	27.5 (21.8–41.8)	27.2 (19.1–38.9)

BMI; body mass index. Age and BMI, are means with range presented in parentheses.

* The number of patients within each groups with samples collected from both blood, muscle and fat.

The study was approved by The Danish Data Protection Agency and the local ethics committee (Region Midtjylland, Denmark number M-20080238 and M-20010248, Central Denmark Regional Committee on Health Research Ethics number 1-10-72-131-15) and registered at ClinicalTrials.gov (NCT00624949, NCT00999310, NCT02526628). All participants provided informed consent.

### Sample preparation, RNA-Seq library construction and sequencing

Blood samples from the antecubital vein were drawn into PAXgene Blood RNA Tubes (Qiagen, Copenhagen, Denmark). Total-RNA was purified using the PaxGene blood Kit 262174 (Qiagen) and the quality was assessed by UV measurements on a Lunatic (Unchained Labs) and on-chip electrophoresis on a Tapestation 4200 RNA Screen Tape System (Agilent, Glostrup, Denmark). Synthesis of directional RNAseq libraries were conducted using the KAPA RNA HyperPrep with RiboErase Globin (HMR) (Roche, Hørsholm, Denmark) following the recommended procedure. Library preparation was automated on a Sciclone NGS (Caliper, Perkin Elmer, Skovlunde, Denmark) liquid handling robot. The quality of the RNA-Seq libraries were estimated by on-chip electrophoresis on a Tapestation 4200 D100 Screen Tape System (Agilent). The library concentrations were estimated using a Qubit dsDNA HS Assay (Thermo Fisher, Roskilde, Denmark). A total of 500 ng RNA was used as input.

Muscle biopsies were taken from the vastus lateralis. The procedure was performed under sterile conditions and local anesthesia (10 ml lidocaine, 10 mg/ml). A small incision was made in the skin parallel to the orientation of the muscle fibers below, and the muscle tissue was extracted using a 5 mm Bergström needle. The biopsies were immediately snap-frozen in liquid nitrogen and stored at -80 °C. RNA was extracted by the RNeasy Fibrous tissue Mini kit (Qiagen). Abdominal subcutaneous fat biopsies were obtained by liposuction technique from the lower abdomen under sterile conditions and local anesthesia (10 ml lidocaine, 10 mg/ml). The biopsies were immediately cleaned, snap-frozen in liquid nitrogen and stored at -80°C. The frozen biopsies were dissociated using a CovarisSP02 (Covaris) and RNA was extracted by the All-prep DNA/RNA/PROTEIN Mini kit 8004 (Qiagen). Synthesis of directional RNA-seq libraries were conducted as described above using the KAPA RNA HyperPrep with RiboErase kit (HMR) (Roche).

The RNA-seq libraries were multiplexed paired-end sequenced on an Illumina Novaseq 6000 (100 bp) and subjected to initial quality control using FastQC (BAbraham Bioinformatics). In addition to trimming of low-quality ends, adaptor removal was conducted using Trim Galore with default settings (BAbraham Bioinformatics).

### CircRNA identification and filtration

CircRNAs were identified from the cleaned fastq files that were obtained as described above. We used a combined approach of three identification pipelines to minimize false positives (Hansen, 2018). These pipelines included CIRI2, CIRCexplorer2 and circRNA_finder (Westholm et al., 2014; Zhang et al., 2016; Gao et al., 2018). CIRI2 was run via CIRIquant using default settings (Zhang J. et al., 2020). For CIRCexplorer2, reads were aligned using STAR and processed using the Parse and Annotate functions (Dobin et al., 2013). The circRNA_finder pipeline was run using default settings for alignment and the minimum length of observed circRNAs was set to 50. CircRNA expression counts from CIRI2 were used for downstream analysis, and only included circRNAs that were also identified by either circRNA_finder or CIRCexplorer2. The circRNA expression was normalized to circular counts per million (circCPM) by dividing raw counts with the total number of circRNAs for each sample, multiplied by one million. A cut-off was applied to minimize the proportion of false positives, leaving only circRNAs expressed by at least 25 circCPM in at least seven samples for further analysis. Circular-to-linear (CTL) ratios were calculated as (2 x circRNA)/(2 x circRNA + linear RNA) as described for CIRI2 (Gao et al., 2018). PAR-regions were assigned to circRNAs with both start and end coordinates within either PAR1 (10,001-2,781,479) or PAR2 (155,701,383-156,030,895). Escape status for X-linked transcripts were collected from Tukiainen et al. (2017). A list of human circRNAs was downloaded from circBase and positions were lifted to hg38 to match our data (Glazar et al., 2014).

### Differential expression of circRNAs

To identify differences in circRNA expression between karyotypes, differential expression analysis was performed by the R-package Limma-Voom using the filtered circRNA raw counts as input (Law et al., 2014). Again, only circRNAs with an expression of 25 circCPM in at least seven samples were included. Limma-Voom was used due to our study design, in order to take the paired nature of samples from each tissue into account. Voom was run for data transformation via the function voomWithQualityWeights due to heterogeneity in the data, and the paired design was taken into account using the duplicateCorrelation function. The batch number was included as a covariate in the design model. Criteria for statistical significance was a Benjamini–Hochberg adjusted *p*-value below 0.05 and an absolute log2 fold change above 1.

### Gene expression estimation and differential gene expression

Gene expression was measured by quasi-mapping using Salmon (Patro et al., 2017). The paired-end fastq files were used as input. A decoy-aware transcriptome index was built based on the hg38 transcriptome, and then, selective alignment was carried out using the fastq pairs as input. Transcript quantities were summarized to gene-level by Tximeta (Love et al., 2020). Differential expression analysis was then carried out using the R Bioconductor package DESeq2 (Love et al., 2014). Only genes with at least 20 counts in more than three samples were included in the analysis. Statistical significance was denoted as a Benjamini–Hochberg adjusted *p*-value below 0.05.

### Prediction of miRNA binding sites and targets and network construction

MiRNA binding sites of selected circRNAs, validated by AGO CLIP-seq, were identified from the web tool Encori StarBase (Li et al., 2014). Only interactions validated by ≥ 2 AGO CLIP-Seq experiments were kept. Experimentally validated microRNA-mRNA interactions were collected from miRTarBase 8.0 (Huang et al., 2020). Interaction networks were constructed using Cytoscape v3.9.0 (Shannon et al., 2003).

## Results

### Dataset description

In order to compare the expression of circRNAs between TS, KS and controls, circRNA transcripts were identified and quantified from whole transcriptome RNA-Seq data obtained from 102 peripheral blood samples, 58 samples of fat and 51 muscle samples. The biopsies were obtained from control males (*n* = 16) and control females (*n* = 34), 47, XXY KS (*n* = 22) and 45,X TS (*n* = 33) (Table 1; Figures 1A–C). The average sequencing depth exceeded 50 million read-pairs per sample (51.7 million read-pairs). To improve circRNA prediction, we used a combined circRNA identification pipeline that included three circRNA identification algorithms (CIRI2, circExplorer2 and circRNA_finder (Figure 1E)). A circRNA was included in this study if it was identified by CIRI2 and at least one of the other circRNA identification algorithms. The circRNA count estimation from CIRI2 was normalized to circular counts per million (circCPM) and lowly expressed circRNAs (<25 circCPM in <7 samples) were removed (Figure 1G), yielding 39,892 circRNAs for downstream analysis (Figure 1D). Of these, 36% had previously been described in the circRNA database circBase.

### Characteristics and genomic origin of circRNAs

**FIGURE 2:**
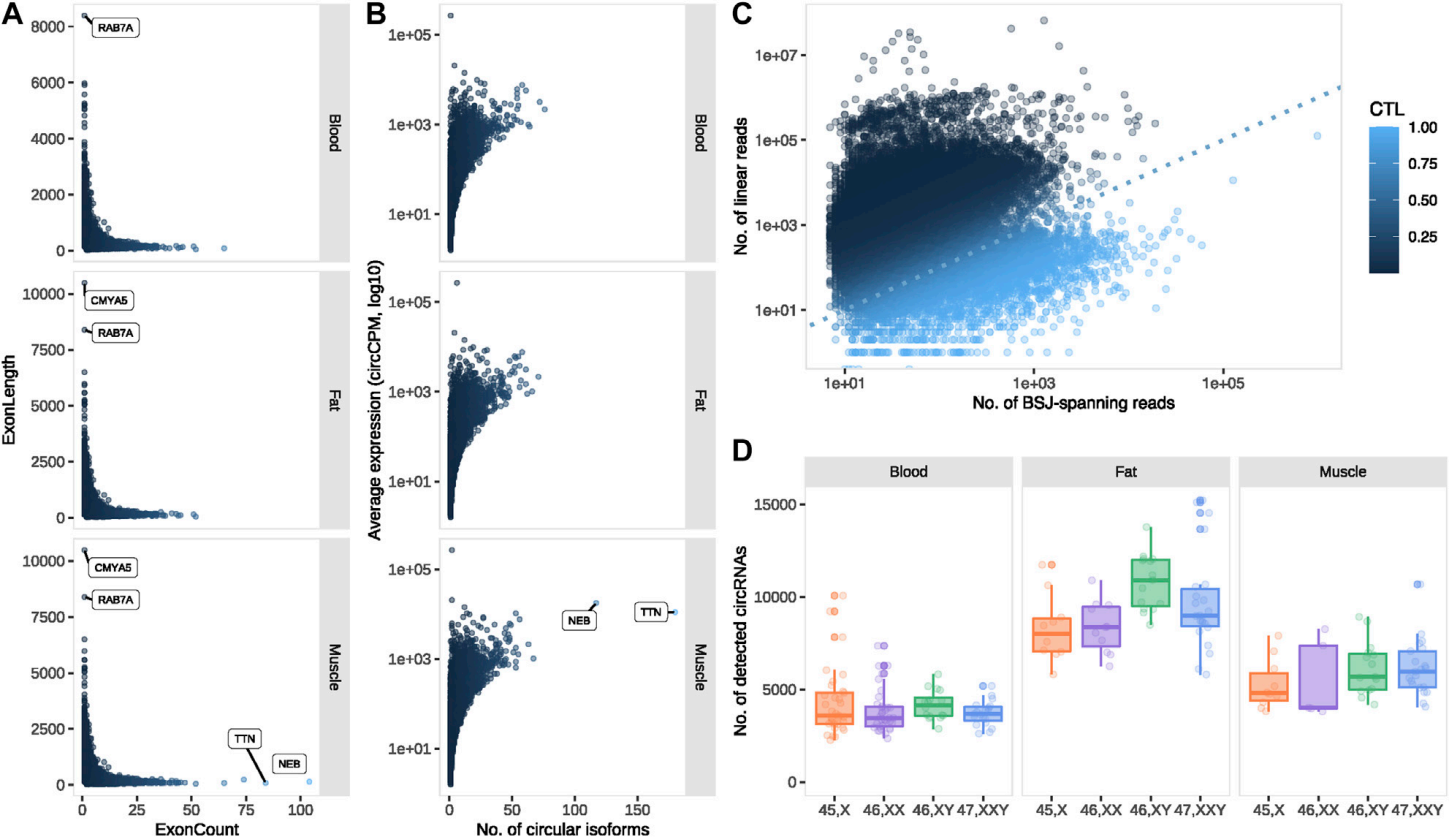
circRNA profiling. **(A)** Exon count vs. length for exonic circRNAs and **(B)** number of circular isoforms vs. average expression for circRNA-producing transcripts, expressed in muscle, blood and fat. **(C)** Circular vs. linear expression for all circRNA-forming transcripts. **(D)** The number of detected circRNAs for each karyotype and tissue. circCPM: circular counts per million. BSJ: back splice junction. CTL: circular-to-linear ratio.

The majority of the circRNAs detected in our dataset were of exonic origin (muscle 86.9%, blood 88.1% and fat 88.9%), and contained multiple exons (average = 5). The highest exon counts were observed in circRNAs from the muscle-specific transcripts encoding Nebulin (*NEB*) and Titin (*TTN*), consisting of 104 and 84 exons, respectively (Figure 2A). We found a general pattern of longer exons from single-exon circRNAs (mean = 767.8 bp), demonstrated by circRNAs from *CMYA5* (chr5:79728914-79739403) and *RAB7A* (chr3:128798036-128806429) that consisted of 10,489 and 8,393 bp, respectively (Figure 2A).

Since a single transcript can give rise to multiple circRNAs, we investigated the number of circular isoforms from each circRNA-forming transcript. The highest number of circular isoforms were observed from the before-mentioned muscle-specific *TTN* (n = 180) and *NEB* (*n* = 117) and were expressed at relatively high levels (average expression; *TTN* = 11098.05 circCPM, *NEB* = 17743.94 circCPM) (Figure 2B). The highest expression was observed from *LINC00632* with regions overlapping *CDR1*, including the well-known and well-characterized circRNA *CDR1as* (average expression 269456.5 circCPM) (Figure 2B) (Hansen et al., 2011).

Circular-to-linear (CTL) ratios were calculated to investigate whether expression was in favor of circular or linear transcripts overlapping the backsplice junction (BSJ) (see Materials and methods). Theoretically, CTL values range from 0, where no circular expression is observed, to one where no linear expression is observed. Among most transcripts (86%), the circRNAs were less abundant than their respective linear counterparts (0 < CTL <0.5) (Figure 2C). However, of the 5,562 (14%) transcripts with higher CTL ratios, 952 had remarkably high ratios (CTL >0.95) and were thus predominately expressed in circular form.

### Tissue and karyotype-dependent circRNA expression patterns

The overall circRNA expression was markedly higher in fat compared to muscle and blood, however, circRNA expression in muscle was also significantly higher compared to blood (*p* < 0.0001 for all tissue comparisons) (Figure 2D). The majority of circRNAs were expressed in all three tissues (64.8%), yet approximately 10% were exclusive to a single tissue. At the tissue level, few circRNAs were absent from fat (8.15%) compared to muscle (16.41%) and blood (19.78%) (Supplementary Figure S1A). By comparison, 87.1% of the circRNAs were expressed in all karyotypes (Supplementary Figure S1B), partly following an anticipated sex-dependent expression due to 54 circRNAs from the Y chromosome, restricted to the 46, XY and 47, XXY samples. Focusing on the 250 most abundant circRNAs in each tissue, only 47 (8.4%) were expressed in all tissues, illustrating a strong tissue-dependency of highly expressed circRNAs (Supplementary Figure S2).

### Differential circRNA expression

**FIGURE 3:**
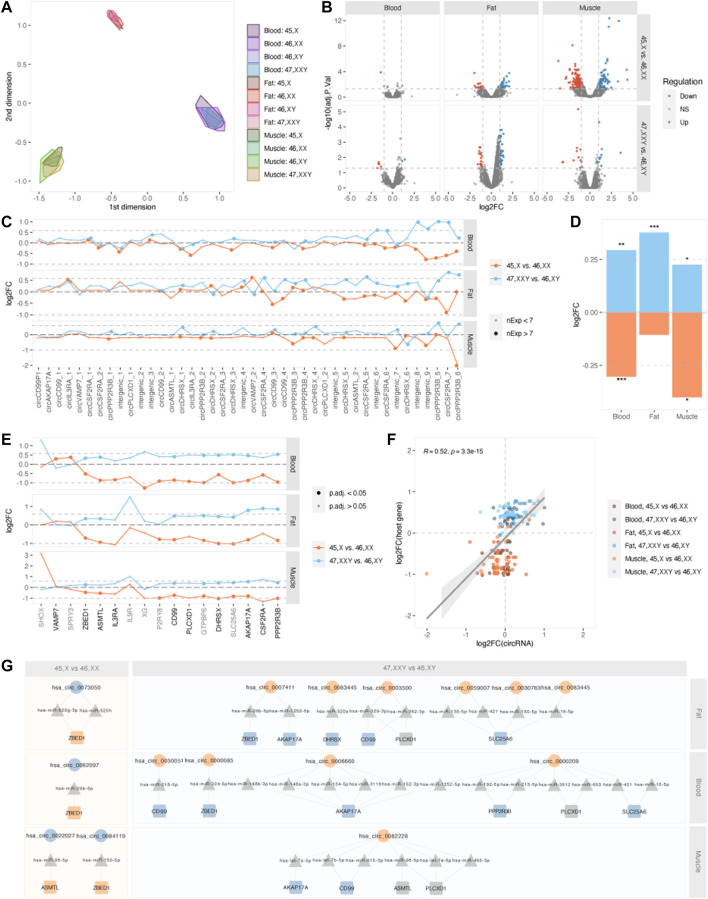
Differential expression and PAR-circRNAs. **(A)** Multidimensional scaling (MDS) of samples, split by tissue and karyotype. **(B)** Volcano plots of–log10 (adjusted *p*-value) and log2FC for all circRNAs in blood (left), fat (middle) and muscle (right), for contrasts 45,X vs. 46, XX (top) and 47, XXY vs. 46, XY (bottom). Upregulation is marked in blue and downregulation in red. **(C)** Log2FCs for PAR-derived circRNAs for contrasts 45,X vs. 46, XX (orange) and 47, XXY vs. 46, XY (blue) in fat (top), blood (middle) and muscle (bottom). Large point size indicates circRNA expression in ≥7 samples within each tissue. Dotted lines indicate the theoretical expressional increase in (log2 (1.5) = 0.585) and decrease (log2 (0.5) = -1) corresponding to one additional or missing X chromosome in 47, XXY vs. 46, XY and 45,X vs. 46,XX. **(D)** Collective log2FC for PAR-derived circRNAs in blood, fat and muscle for the contrasts 45,X vs. 46, XX (orange) and 47, XXY vs. 46, XY (blue). *p < 0.05, **p < 0.01, ***p < 0.001, one-sample *t*-test for each comparison. **(E)** Log2FCs for PAR-genes for contrasts 45,X vs. 46, XX (orange) and 47, XXY vs. 46, XY (blue) in fat (top), blood (middle) and muscle (bottom). PAR-genes are marked in black writing if they form circRNA and grey if not. Point size indicates gene significance, a large dot indicating an adjusted *p*-value below 0.05. Dotted lines indicate the theoretical expressional increase in (log2 (1.5) = 0.585) and decrease (log2 (0.5) = -1) corresponding to one additional or missing X chromosome in 47, XXY vs. 46, XY and 45,X vs. 46,XX. **(F)** Correlation of log2FCs for PAR-derived circRNAs and host genes. Tissues and contrasts are specified by color. **(G)** Potential circRNA (circles)-miRNA (triangles)-mRNA (squares) networks affecting PAR-genes. Up- and downregulated circRNAs and genes are marked as blue and orange shapes. 45,X vs. 46, XX is marked in orange and 47, XXY vs. 46, XY in blue squares.

Next, we identified differences in circRNA expression in TS and KS compared to female and male controls. We did not observe a pronounced difference among circRNA expression profiles between karyotypes, yet expression patterns clearly differentiated based on tissue of origin (Figure 3A). Differentially expressed circRNAs (DECs) (p.adj <0.05 and |log2FC|>1) were identified in both 45,X vs. 46, XX and 47, XXY vs. 46, XY in all tissues. In general, few DECs were observed in blood (47, XXY vs. 46,XY, n = 8, 45,X vs. 46,XX, n = 3) compared to muscle (47, XXY vs. 46,XY, n = 19, 45,X vs. 46,XX, n = 305) and fat (47, XXY vs. 46,XY, n = 53, 45,X vs. 46,XX, n = 52) (Supplementary Table S1, Figure 3B), indicating predominantly karyotype- and sex-independent circRNA expression patterns in blood. No overlapping DECs between the comparisons 45,X vs. 46, XX and 47, XXY vs. 46, XY were present in blood or fat, while two were found in muscle, spanning Myosin Heavy Chain (MYH1/2) (chr17:10505811-10533632) as well as *NEB* (chr2:151494160-151497033).

To investigate if circRNAs were affected directly by X chromosomal dosage, we singled out circRNAs that derived from PAR. These included circRNAs arising from the PAR-genes *AKAP17A*, *ASMTL*, *CD99*, *CD99P1* (pseudogene), *CSF2RA*, *DHRSX*, *PLCXD1*, *PPP2R3B* and *VAMP7* as well as intergenic segments within PAR regions (Figure 3C). We observed a collective upregulation of PAR1-derived circRNAs in KS compared to males and downregulation in TS compared to females (Figure 3D). These comparisons were statistically significant in all tissues, except for fat X vs. XX (one-sample *t*-test, blood X vs. XX: logFC = -0.304, *p* = 9.75E-05; blood XXY vs. XY: logFC = 0.294, *p* = 0.0087; muscle X vs. XX: logFC = -0.401, *p* = 0.015; muscle XXY vs. XY: logFC = 0.225, *p* = 0.029; fat X vs. XX: logFC = -0.105, *p* = 0.179; fat XXY vs. XY: logFC = 0.378, *p* = 1.95E-05). By incorporating gene expression data, the same pattern was also observed for the circRNA-forming PAR genes themselves (Figure 3E, black font). Here, we observed a general up-regulation when comparing 47, XXY with 46, XY and downregulation when comparing 45,X with 46, XX (Supplementary Table S2). With few exceptions, this was evident for all PAR genes, in all tissues. We further investigated the correlation between log2 fold changes of PAR-circRNAs and their host genes, which revealed a general down-regulation in 45,X vs. 46, XX and general up-regulation in 47, XXY vs. 46,XY. Interestingly, we observed a more pronounced X-dosage effect on mRNA expression compared to circRNAs (Figure 3F).

Considering all three tissues, the most X-dosage sensitive circRNAs were chrX:338603-347693 and chrX:1285777-1290509, derived from *PPP2R3B* and *CSF2RA*. Investigating the expression levels of these circRNAs, we observed tissue dependency (Supplementary Figure S3). The classical dosage stoichiometry of sex chromosome counts 1:2:2:3 for X:XY:XX:XXY was not obvious, yet a tendency was observed. *circPPP2R3B* was expressed in muscle samples of all karyotypes except for 45,X (Supplementary Figure S2A), and *circCSF2RA* in samples of blood and fat (Supplementary Figure S3B). In addition to this, many PAR transcripts displayed a distinct all-or-nothing pattern of CTL values being either zero or one, showing that expression was only present in some samples and, if present, that the respective transcript only existed in either circular or linear form (Supplementary Figure S4).

### Proposed regulatory circRNA-miRNA-mRNA networks

CircRNAs may function as competing endogenous RNAs (ceRNAs), regulating gene expression via miRNA-sponging. Thus, miRNAs with potential binding sites in DECs with known circBase-IDs, were obtained from the ENCORI StarBase (miRNA-circRNA interactions supported by Ago CLIP-Seq data). CircRNA-miRNA Interactions validated by ≥ 2 AGO CLIP-Seq experiments were included, and the target genes of these miRNAs were obtained from the experimentally validated miRNA-target interactions database miRTarBase (Figures 1E, F). From these target genes, only differentially expressed genes (DEGs) identified from the 45, X vs. 46, XX and 47, XXY vs. 46, XY comparisons and PAR genes expressed in our data were kept for network construction (Figures 1F–H).

As most PAR genes escape XCI, we would expect an expressional increase in KS (log2FC 0.585) and decrease in TS (log2FC = -1), if these genes followed dosage stoichiometry. However, this is not the case for all PAR-genes (Figure 3E). Therefore, we speculated if circRNAs regulate PAR gene expression by exerting a modifying effect, possibly through miRNA-sponging. In theory, this would mean downregulation of certain circRNAs in KS that led to a release of miRNAs that target PAR genes. In TS, the opposite scenario should result in an increase in PAR gene expression. Based on DECs from both 45,X vs. 46, XX and 47, XXY vs. 46, XY in all tissues, we identified several circRNA-miRNA-mRNA networks that could cause this regulation (Figure 3G). For 45,X vs. 46, XX we observed a log2FC of approximately -0.5 for *ZBED1* in all tissues, corresponding to only a 50% downregulation compared to the expected 100% for PAR genes escaping XCI. This could be caused by up-regulated circVDAC3 (hsa_circ_0084119), circPCNT (hsa_circ_0062097) and circCOL4A3BP (hsa_circ_0073050), sponging miRNAs hsa-miR-150-5p, hsa-miR-26b-5p, hsa-miR-520g-3p and hsa-miR-520 h. Similarly, a 50% upregulation of *ZBED1* was observed in 47, XXY vs. 46, XY for all tissues, with circRNA implication in blood and fat from circANKRDD13C (hsa_circ_0000085) and circPTPN12 (hsa_circ_0007411) sponging hsa-miR-324-5p and hsa-miR-20b-5p. The same pattern was observed for *ASMTL* in muscle, potentially caused by upregulation of circMTCH2 (hsa_circ_0022027) in 45,X vs. 46, XX and downregulation of circFLNC (hsa_circ_0082228) in 47, XXY vs. 46,XY, both sponging hsa-miR-98-5p in muscle. Based on these interactions, we hypothesize that regulation of the PAR1 genes *ASMTL* and *ZBED1* could be associated to circRNAs, decreasing downregulation in TS and upregulation in KS, possibly as a compensatory mechanism.

**FIGURE 4:**
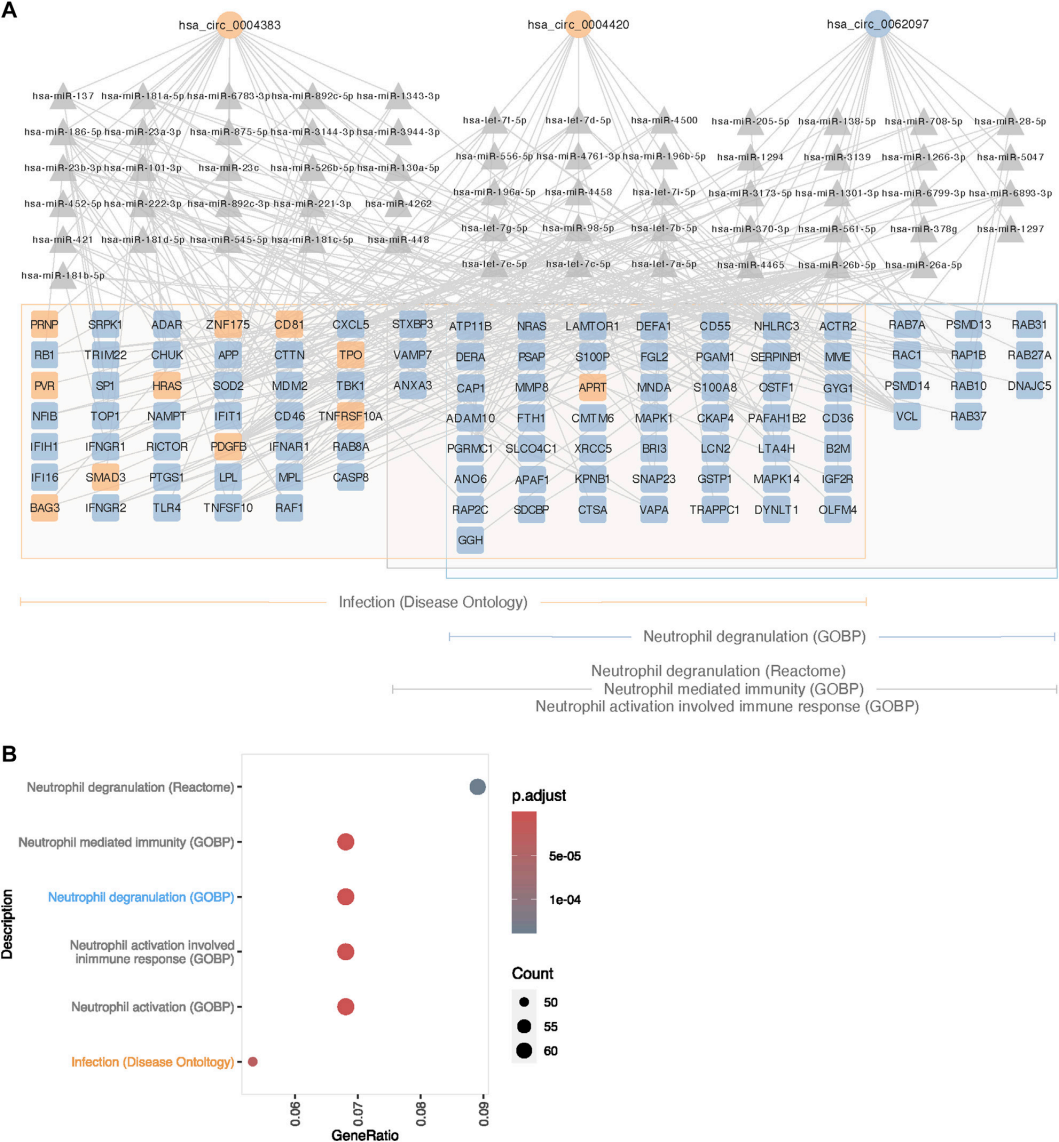
Proposed circRNA-miRNA-mRNA network affecting immune function in 45,X blood. **(A)** Differentially expressed circRNAs (circle), predicted miRNA targets supported by ≥ 2 AGO CLIP-seq experiments (traingle) and potential mRNA targets (square), differentially expressed and enriched for immune functions. Up- and downregulated circRNAs and mRNAs are colored in blue and orange. Framed genes are matched to their respective affiliation to ontologies related to immune functions; orange for Infection (Disease Ontology), blue for Neutrophil Degranulation (Gene Ontology Biological Process) and grey for Neutrophil Mediated Immunity (Gene Ontology Biological Process), Neutrophil Activation Involved Immune Response (Gene Ontology Biological Process) and Neutrophil Degranulation (Reactome). **(B)** Immune system-related gene ontology and pathway enrichment for differentially expressed genes in the 45,X vs. 46, XX contrast in blood, targeted by miRNAs predicted to interact with differentially expressed circRNAs.

To analyze if the circRNAs regulate gene expression elsewhere in the genome through miRNA-sponging, we manually constructed networks of DECs, miRNAs and target DEGs, and found that several DECs interact with miRNAs that had DEGs as predicted targets. Enrichment analysis was performed on DEGs within each of these networks, revealing enrichment of pathways and terms associated with clinical traits of TS and KS (Supplementary Figures S5, 6). Of these, terms relating to the immune system occurred frequently in the 45,X vs. 46, XX contrast in blood (Figure 4A). Enriched terms within this description included the disease ontology “Infection” as well as one Reactome pathway and four GO Biological processes related to neutrophils. 105 DEGs were included in these terms, of which the vast majority were upregulated (94/105) in the 45,X vs. 46, XX contrast. These genes were predicted to interact with 60 miRNAs, interacting with three circRNAs (Figure 4B), circZNF292 (hsa_circ_0004383), circTRABD2A (hsa_circ_0004420) and circPCTN (hsa_circ_0062097). All but one gene associated with neutrophils (*APRT*) were upregulated, potentially caused by miRNA sponging by the upregulated circRNA hsa_circ_0062097.

### CircRNAs associated with phenotypic traits

**FIGURE 5:**
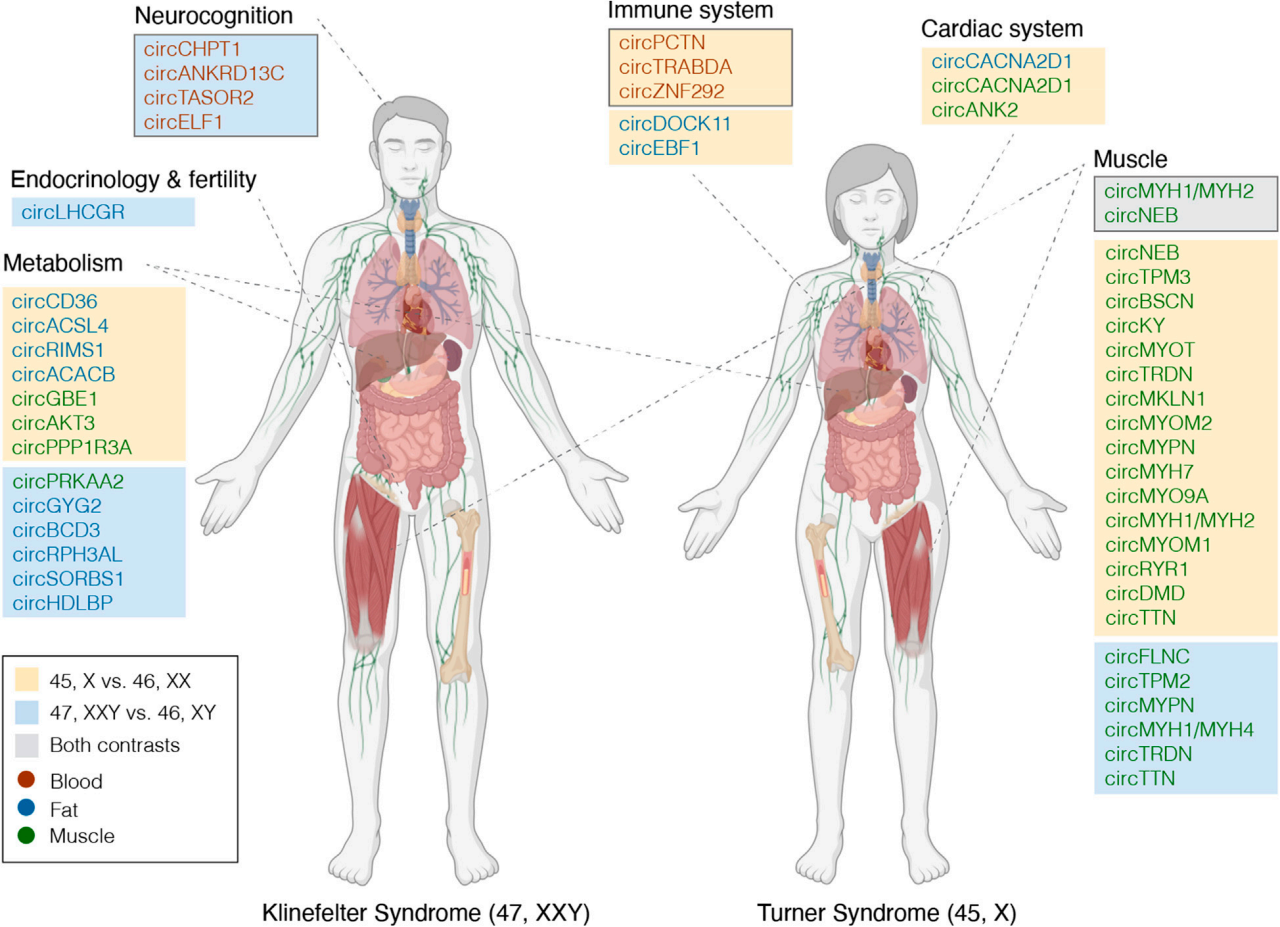
Tentative candidate circRNAs functionally linked to TS and KS phenotypes. circRNAs potentially involved in the KS and TS phenotypes, appointed to phenotypic traits based on circRNA-miRNA-mRNA networks and enrichment analyses (framed boxes a-b) or host gene functionality. Immune system, framed box contains circRNAs differentially expressed in blood for contrast 45,X vs. 46,XX, potentially affecting expression of genes related to immune functions. Neurocognition, framed box contains circRNAs differentially expressed in blood for contrast 47, XXY vs. 46,XY, potentially affecting expression of genes related to neurocognition. Muscle, framed box contains circRNAs differentially expressed in muscle for both contrasts. circRNAs are marked by significance; orange, blue or gray boxes if significant in 45,X vs. 4,6XX, 47, XXY vs. 46, XY or both. Tissue is specified by text color as presented in the legend. Part of this figure was created with BioRender.com.

As observed from the enrichment analysis based on genes from the circRNA-miRNA-mRNA networks that were identified in the previous section, three DECs could theoretically alter gene expression in blood cells from TS females, that had a relation to pathways involved in immune, and particularly, neutrophil functions (Figure 5), thus contributing to the increased risk of autoimmune disease and minor immune deficiencies (Jorgensen et al., 2010; Bakalov et al., 2012). Likewise, in blood cells from KS males, enriched disease ontologies were related to cognition (Supplementary Figure S6), and our analysis revealed four DECs (circCHPT1 (hsa_circ_0006660), circANKRD13C (hsa_circ_0000085), circTASOR2 (hsa_circ_0000209), circELF1 (hsa_circ_0030051)) implicated in regulating genes involved in these neurological traits (Figure 5).

Moreover, circRNAs are believed to be related to their respective host gene through competition with linear splicing or through regulatory mechanisms. (Holdt et al., 2018). Hence, the function of these host genes is of interest to disclose the impact of DECs. Interestingly, the host genes of many DECs could be related to specific TS and KS phenotypic traits (Figure 5, unmarked boxes). DECs derived from muscle originated from host genes related to muscle function. For example, 23 DECs in 45,X vs. 46, XX originated from the gene *TTN*, yet the host gene itself showed no differential expression (log2FC 0.19, p. adj. 0.55). Alongside a circRNA spanning parts of *MYH1* and *MYH2*, these were the only DECs common between both contrasts (Figure 5). From muscle and fat, several host genes from both contrasts were related to metabolism, and specifically to fatty acids, lipoproteins, glycogen and insulin (e.g., *ACSL4*, *GBE1*, *HDLBP*, *SORBS1*). For 45,X vs. 46,XX, the host gene functions in muscle could be related to the cardiac system, with three DECs derived from *ANK2*. Lastly, one DEC of particular interest from 47, XXY vs. 46, XY derived from *LHCGR,* functioning as the receptor for luteinizing hormone.

## Discussion

While the genetic mechanism underlying the phenotype of sex chromosome abnormalities such as TS and KS is still not understood, the implication of fundamental transcriptional and epigenetic changes has been widely demonstrated (Trolle et al., 2016; Skakkebaek et al., 2018; Zhang X. et al., 2020). Expressional and methylation changes are not constricted to protein coding genes, but also involve the class of non-coding RNAs that control an underlying regulatory network not yet disclosed in these syndromes. One factor of such networks, ignored in ordinary transcriptional analysis, is circRNAs, which are characterized by regulatory potency, high stability and tissue specificity.

Here, we used a combination of three circRNA identification pipelines to robustly identify circRNAs from transcriptional data of several tissues of interest and carried out a comparative analysis of the circular transcriptomes of TS and KS. A multi-tissue setup was used, as each tissue will have its own specific gene expression profile. Blood, fat and muscle tissue was chosen due to their biological relevance in these syndromes and accessibility. Relevant tissues for both Turner and Klinefelter syndrome are tissues where the two syndromes are known to play a role. Such tissues include the gonads, where a gonadal dysgenesis takes place in both syndromes, and the brain where both syndromes are characterized by neurocognitive changes. However, both these tissues are difficult to obtain. Meanwhile, both syndromes are also characterized by an increased frequency of the metabolic syndrome, type 2 diabetes and other metabolic changes, and therefore both fat and muscle can be seen as target tissues for this part of the phenotype. These tissues are more easily accessible. In addition, we chose also to sample blood, in order to catch a glimpse of the differences and similarities across these three tissues. Furthermore, the current study populations of TS and KS patients are comparable to their control counterparts in terms of hormone levels, because all TS and KS were treated with their respective missing hormones (i.e. Estradiol and testosterone), and therefore, the differences we observe should reflect the actual differences between the syndromes and their controls and not be affected in any major way by differences in sex hormones.

We identified approximately 40,000 circRNAs in our dataset and these circRNA showed a highly tissue-dependent expression pattern, as reported in previous studies in the general population (Xia et al., 2017). DECs were detected genome-wide in both TS and KS in all tissues, showing that the circular transcriptome is also impacted in SCAs. The DECs were derived from both the autosomes and the X chromosome, indicating that non-euploid sex chromosome numbers affect the entire circular transcriptome.

As expected, we observed that circRNAs from the PAR1 region were predominately downregulated in TS and upregulated in KS relative to their controls. Supporting this, circRNAs derived from the PAR2 region, with missing XCI escape status, did not follow this pattern. This showed that circRNAs are sensitive to X chromosome dosage and dependent on the X inactivation pattern. However, few of these circRNAs passed statistical significance as differentially expressed, and none overlapped the two contrasts, 45,X vs. 46, XX and 47, XXY vs. 46,XY. Furthermore, we found that the X chromosome dosage dependency was more pronounced on the mRNA than the circRNA level. Upon further inspection we observed that most PAR1 circRNAs were not expressed in all samples indicating that the expression of circRNAs is less pervasive than that of their host genes, which could be due to a more dynamic nature of the underlying regulatory mechanisms of circRNAs. This limited pervasiveness was further demonstrated by the circular-to-linear ratios, as the circular form was rarely equally or more expressed than their linear counterpart. Nevertheless, the general expression patterns of some PAR1-derived circRNAs did follow X dosage stoichiometry (1:2:2:3; 45,X:46,XY:46,XX:47,XXY). The expressional directionality of these PAR1-circRNAs provides a possibility that circRNAs could be involved in specific inversely correlated traits of TS and KS.

As circRNAs are commonly known to regulate transcription through miRNA sponging, we aimed to investigate potential circRNA-miRNA-mRNA networks in relation to comorbidities and phenotypic traits in TS and KS. Only two studies have investigated miRNAs in SCAs, and both were performed on PBMC of KS individuals and male controls (Sui et al., 2012; Cimino et al., 2017). Because of the unavailability of TS and KS specific miRNA datasets, circRNA-miRNA-mRNA networks were constructed based on experimentally validated interactions obtained from publicly available databases. Moreover, these miRNA interactions only included circRNAs with known circBase IDs, possibly leaving out essential circRNA-miRNA interactions involving circRNAs not reported in circBase. Our findings suggested that several differentially expressed circRNAs have the potential to affect PAR1 genes through interactions with miRNAs. This included regulatory mechanisms for the PAR1 genes *ZBED1* and *ASMTL*, both of which displayed less X chromosome dosage sensitivity than expected. This implies that these circRNAs were responsible for toning down the expected expressional changes of *ZBED1* and *ASMTL*. While the transcription factor *ZBED1* has been reported to be involved in proliferation and viral infection, little is known about *ASMTL* (Yamashita et al., 2007; Radko et al., 2014).

In TS, enrichment analysis using differentially expressed genes that were part of the identified circRNA-miRNA-mRNA networks as input, revealed enrichment within immune functions from blood. Here, we present a network of three circRNAs, 60 miRNAs and 105 mRNAs. Almost all these 105 mRNAs were upregulated and were associated with neutrophil functionality in TS compared to euploid control females. Within the innate immune system, inflammation and autoimmune diseases, neutrophils are of particular interest in TS. TS shows increased levels of pro-inflammatory factors IL6 and TGF b1 and decreased levels of anti-inflammatory factors IL10 and TGFb2, (Bakalov et al., 2012), as well as an increased incidence of autoimmune diseases, especially inflammatory bowel disease and thyroiditis. (Lleo et al., 2012). The neutrophils are the most abundant leukocyte, and thus, we find it interesting that these immune related findings were only observed in blood. Moreover, no dysregulated pattern of inflammation has been reported in KS, and supporting this, no enrichment of terms related to neutrophil functionality an immune system from circRNA-miRNA-mRNA networks were obtained.

In KS, a similar enrichment analysis approach revealed that differentially expressed genes from circRNA-miRNA-mRNA networks were related to the disease ontologies “Mental Retardation, X-Linked” and “Severe psychomotor retardation”. Supporting this, the KS phenotype has been shown to be associated with deficits in language, intelligence and executive functions (Gravholt et al., 2018). This enrichment analysis further found terms such as “Turner syndrome”, “XX males” and “Gonadal dysgenesis, 45,X” to be associated with genes within the network from KS blood, indicating commonality between multiple sex chromosome abnormalities. These genes included XIS*T*, *SMC1A*, *RPS4X*, *PRKX*, *ZFX*, *CD99* and *KDM6A*, all of which appear on the X chromosome and either escape XCI, are involved in XCI or have homologs on the Y chromosome.

In both TS and KS, several differentially expressed circRNAs arose from genes functionally linked to phenotypic traits of these syndromes. Interestingly, the only circRNAs that were differentially expressed in both TS and KS, compared to their respective controls, were both downregulated and derived from the muscle restricted transcripts *TTN* and *MYH1/2*. Alongside these, several other circRNAs derived from either these or other muscle related host genes were differentially expressed in either TS or KS. Both TS and KS have been associated with phenotypes of decreased muscle mass and strength (Gravholt et al., 2006; Gravholt et al., 2018) and circRNAs have been reported as associated with myogenesis and muscle disease such as myotonic dystrophy (Legnini et al., 2017; Voellenkle et al., 2019). Similarly, both TS and KS are associated with increased weight, altered body composition and metabolic disease, including diabetes (Gravholt et al., 2018; Gravholt et al., 2019) and we found that several DECs were from host genes related to metabolism. Of special interest is the DEC from 47, XXY vs. 46, XY fat originating from *SORBS1*, which is involved in insulin-stimulated glucose transport (Saltiel and Kahn, 2001). Furthermore, one DEC from 45,X vs. 46, XX in fat, which is derived from *ACSL4*, may be involved in fatty acid metabolism and metabolic syndrome (Zeman et al., 2009). A specific circRNA derived from *LHGCR* is of interest due to previous studies revealing importance for reproductive physiology, as mutations caused cryptorchidism, immature Leydig cells and hypergonadotropic hypogonadism, all of which are traits of KS.

## Conclusion

In conclusion, the present study shows changes in the circRNA transcriptome of TS and KS throughout three different tissues. Thus, this study extends our current understanding of the genomics behind the phenotype of TS and KS and goes beyond the previously reported changes in the mRNA transcriptome and methylome. The conceptual understanding of disease mechanisms in these syndromes is suggestibly much more complex than hitherto thought. We therefore propose that the phenotype of individual patient with TS and KS is a result of multiple regulating genomic mechanisms, acting through an array of complicated multi-tissue frameworks to be further investigated.

## Data Availability

The data presented in the study are deposited in the EGA repository, accession number EGAS00001006404.
