# Supplementary material for: Sex chromosome aneuploidies give rise to changes in the circular RNA profile: A circular transcriptome-wide study of Turner and Klinefelter syndrome across different tissues
Source: Front Genet. 2022 Jul 22;13:928874. doi: 10.3389/fgene.2022.928874 (PMC9355307; doi:10.3389/fgene.2022.928874)
Supplement: Supplementary file 1 [file DataSheet1.docx]

Supplementary Material

## Supplementary Figures

**Supplementary Figure 1.** Overlaps in detected circRNAs based on A) tissue and B) karyotype.


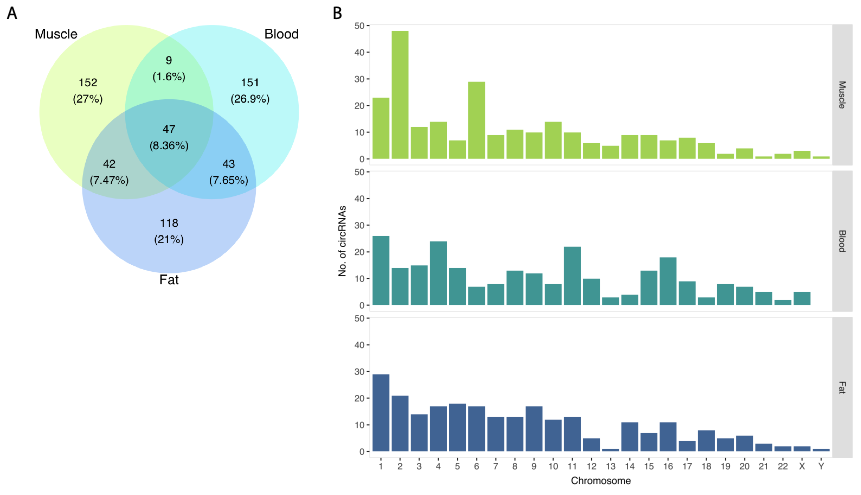


**Supplementary Figure 2.** Top 250 most abundant circRNAs in each tissue. A) Overlaps in abundant circRNAs from each tissue. B) Chromosomal origin for abundant circRNAs from each tissue.

**Supplementary Figure 3.** Expression levels of selected PAR-circRNAs.
Expression levels (circular counts per million, circCPM) in blood, fat and muscle from 45,X, 46,XX, 46,XY and 47,XXY of circRNAs (A) circPPP2R3A and (B) circCSF2RA, originating from the pseudoautosomal region 1 (PAR1).

**Supplementary Figure 4.** CTLs of selected PAR-circRNAs.
Circular-to-linear (CTL) ratios of circRNAs (A) circPPP2R3A and (B) circCSF2RA, originating from the pseudoautosomal region 1 (PAR1) in blood, fat and muscle from 45,X, 46,XX, 46,XY and 47,XXY. Each dot represents the CTL for one individual.

**Supplementary Figure 5.** Disease ontology (a), gene ontology (b) and pathway enrichment (c) analysis for differentially expressed genes in the 45,X vs. 46,XX contrast in blood, targeted by miRNAs predicted to interact with differentially expressed circRNAs.

**Supplementary Figure 6.** Disease ontology enrichment analysis for differentially expressed genes in the 47,XXY vs. 46,XY contrast in blood, targeted by miRNAs predicted to interact with differentially expressed circRNAs.

**Supplementary Table 1. Differentially expressed circRNAs.**

|  | logFC | CI.L | CI.R | AveExpr | P.Value | adj.P.Val |
| --- | --- | --- | --- | --- | --- | --- |
| **Blood** | | | | | | |
| *KS (47,XXY) vs. Male (46,XY)* | | | | | | |
| chr1:229525935-229549434 | 1.1035 | 0.85568 | 1.3513 | 4.9738 | 3.8650e-06 | 0.017132 |
| chr9:123757702-123879021 | 1.4021 | 1.18206 | 1.6221 | 5.8151 | 2.5692e-06 | 0.013632 |
| chr10:5699524-5714207 | -1.6430 | -1.90799 | -1.3780 | 7.1451 | 5.1606e-06 | 0.020587 |
| chr8:23109544-23111311 | -1.6461 | -1.86708 | -1.4251 | 5.7527 | 7.5182e-06 | 0.027033 |
| chr7:93291702-93297243 | 1.0060 | 0.65224 | 1.3598 | 5.3159 | 2.7483e-05 | 0.049835 |
| chr13:40940920-40943925 | -1.5789 | -1.88653 | -1.2714 | 6.9878 | 8.8094e-06 | 0.027033 |
| chr1:70292387-70315566 | -1.3656 | -1.62073 | -1.1104 | 7.2005 | 1.5027e-05 | 0.039965 |
| chr12:101714089-101716812 | -1.7837 | -2.23389 | -1.3336 | 6.6234 | 2.5367e-05 | 0.049835 |
| *TS (45,X) vs. Female (46,XX)* | | | | | | |
| chr6:87210450-87218731 | -1.3937 | -1.60095 | -1.18644 | 7.6300 | 3.2353e-09 | 0.000129 |
| chr21:46363479-46367139 | 1.0861 | 0.89399 | 1.27825 | 5.4749 | 1.7140e-06 | 0.015874 |
| chr2:84870224-84870785 | -1.0061 | -1.18858 | -0.82353 | 5.2839 | 2.7708e-06 | 0.018422 |
| **Fat** | | | | | | |
| *KS (47,XXY) vs. Male (46,XY)* | | | | | | |
| chr2:39325517-39326277 | 1.2087 | 0.709790 | 1.70770 | 4.8060 | 5.9703e-09 | 0.000158 |
| chr18:32111753-32129327 | 1.0338 | 0.772609 | 1.29505 | 4.7520 | 7.9344e-09 | 0.000158 |
| chr2:48694223-48714054 | 1.4032 | 1.084468 | 1.72187 | 4.8606 | 2.1258e-08 | 0.000282 |
| chr1:32224033-32224475 | 1.0845 | 0.223379 | 1.94565 | 4.7387 | 4.6069e-08 | 0.000367 |
| chr10:37218979-37219897 | 1.1241 | 0.051123 | 2.19710 | 4.8607 | 1.1228e-07 | 0.000430 |
| chr8:16710237-16711434 | 1.0071 | 0.597912 | 1.41635 | 4.7308 | 1.3996e-07 | 0.000430 |
| chr8:6508931-6513844 | 1.0241 | 0.623877 | 1.42439 | 4.7387 | 1.5812e-07 | 0.000430 |
| chrX:2853979-2881224 | 1.0114 | 0.421254 | 1.60154 | 4.7661 | 2.3510e-07 | 0.000493 |
| chr1:27749067-27755330 | 1.0126 | 0.626464 | 1.39876 | 4.7746 | 3.0855e-07 | 0.000559 |
| chr7:11022867-11051780 | 1.0666 | 0.747792 | 1.38544 | 4.7648 | 5.4348e-07 | 0.000867 |
| chr1:219210531-219241308 | 1.2501 | 0.870348 | 1.62995 | 4.9063 | 1.3372e-06 | 0.001367 |
| chr4:171121511-171147972 | -1.2807 | -1.794449 | -0.76696 | 4.9021 | 3.1479e-06 | 0.002176 |
| chr11:75851882-75961549 | -1.3797 | -1.688614 | -1.07072 | 4.9026 | 3.1644e-06 | 0.002176 |
| chr14:75087017-75088641 | 1.1411 | 0.722792 | 1.55942 | 4.8876 | 6.9690e-06 | 0.003432 |
| chr5:96873285-96873672 | -1.4136 | -1.749972 | -1.07726 | 5.2391 | 9.1524e-06 | 0.003803 |
| chr4:152382188-152412529 | 1.1533 | 0.662811 | 1.64383 | 4.8913 | 1.2534e-05 | 0.004347 |
| chr4:129039040-129082373 | 1.1934 | 0.787212 | 1.59954 | 4.9749 | 1.2144e-05 | 0.004249 |
| chr11:14771936-14869630 | 1.3484 | 1.033121 | 1.66376 | 5.0828 | 8.8498e-06 | 0.003803 |
| chr4:26748410-26769852 | 1.1147 | 0.210486 | 2.01883 | 4.8777 | 1.6287e-05 | 0.005115 |
| chr14:35096557-35108236 | 1.0079 | 0.712462 | 1.30328 | 4.8336 | 1.8821e-05 | 0.005582 |
| chr16:49822669-49827083 | -1.1996 | -1.512997 | -0.88620 | 4.9362 | 1.5920e-05 | 0.005080 |
| chr7:22266963-22318037 | -1.4356 | -2.503675 | -0.36757 | 5.0605 | 1.8427e-05 | 0.005582 |
| chr4:99279425-99307864 | 1.3571 | 0.960207 | 1.75400 | 4.9300 | 3.2652e-05 | 0.007358 |
| chr3:111897705-111945357 | 1.0683 | 0.648270 | 1.48839 | 4.8955 | 3.1647e-05 | 0.007186 |
| chr18:54155237-54166166 | -1.2245 | -1.514488 | -0.93443 | 5.0030 | 2.5401e-05 | 0.006537 |
| chr18:79154503-79214038 | 1.2274 | 0.829884 | 1.62497 | 5.7704 | 3.4846e-05 | 0.007554 |
| chr14:30705822-30734858 | -1.0296 | -1.370941 | -0.68832 | 4.8822 | 4.2713e-05 | 0.008238 |
| chr1:94458606-94483239 | 1.4136 | 0.993224 | 1.83401 | 5.0781 | 5.8364e-05 | 0.009239 |
| chr2:99438600-99464985 | 1.0674 | 0.678047 | 1.45669 | 4.8760 | 9.8412e-05 | 0.011879 |
| chr17:281767-327579 | 1.0533 | 0.741222 | 1.36539 | 4.9138 | 9.5753e-05 | 0.011825 |
| chr19:42977462-42995361 | -1.2157 | -1.629549 | -0.80190 | 4.9923 | 8.5254e-05 | 0.010847 |
| chr22:45330602-45332710 | 1.2707 | 0.907122 | 1.63419 | 5.0863 | 1.4861e-04 | 0.015318 |
| chr2:62826086-62874532 | 1.0344 | -0.111200 | 2.17998 | 5.0239 | 1.6209e-04 | 0.016005 |
| chr4:145870244-145903215 | 1.1638 | 0.764910 | 1.56267 | 4.9743 | 1.7930e-04 | 0.016931 |
| chr15:49632158-49634794 | 1.1061 | 0.817182 | 1.39509 | 4.9476 | 2.3264e-04 | 0.019497 |
| chr10:95339132-95376056 | -1.1969 | -1.638619 | -0.75510 | 5.0865 | 1.9494e-04 | 0.017754 |
| chr1:117460488-117522937 | 1.0852 | 0.707577 | 1.46288 | 5.1736 | 2.9838e-04 | 0.022586 |
| chr8:17653185-17656065 | 1.0628 | 0.750981 | 1.37459 | 5.0421 | 3.0380e-04 | 0.022823 |
| chr11:36610080-36648155 | 1.0197 | 0.620628 | 1.41874 | 4.9755 | 3.1963e-04 | 0.023430 |
| chr18:62544801-62556297 | 1.1345 | 0.796922 | 1.47207 | 6.2555 | 4.1694e-04 | 0.026833 |
| chr7:77581426-77600806 | -1.2799 | -1.611735 | -0.94812 | 5.2210 | 3.8796e-04 | 0.026186 |
| chr6:143772257-143774215 | 1.1803 | 0.082869 | 2.27764 | 5.0440 | 4.2568e-04 | 0.026996 |
| chr12:1115865-1190052 | -1.2057 | -1.512715 | -0.89876 | 5.1839 | 4.0306e-04 | 0.026576 |
| chr8:17753716-17755961 | -1.1941 | -1.520367 | -0.86792 | 5.7760 | 4.3258e-04 | 0.027347 |
| chr9:112268048-112297916 | -1.5724 | -1.952496 | -1.19222 | 5.6327 | 4.5044e-04 | 0.028076 |
| chr2:201545542-201582217 | 1.0436 | 0.710794 | 1.37642 | 5.0421 | 5.6471e-04 | 0.031684 |
| chr16:19733697-19735450 | 1.0201 | 0.670174 | 1.37009 | 4.9161 | 6.2304e-04 | 0.033316 |
| chr9:95966561-96004701 | -1.2204 | -1.661977 | -0.77890 | 5.0659 | 6.2710e-04 | 0.033323 |
| chr7:11244257-11322424 | -1.0531 | -1.438553 | -0.66760 | 5.2679 | 6.1369e-04 | 0.033038 |
| chr19:42994749-42995361 | -1.1336 | -1.460875 | -0.80634 | 5.0644 | 6.8681e-04 | 0.035216 |
| chr2:241229832-241249980 | -1.1152 | -1.421657 | -0.80883 | 5.0165 | 9.6097e-04 | 0.042452 |
| chr6:82957311-83038485 | 1.0426 | 0.699615 | 1.38552 | 4.9747 | 1.2436e-03 | 0.049173 |
| chr13:100301459-100340259 | -1.4358 | -1.756760 | -1.11484 | 5.6175 | 1.2830e-03 | 0.049934 |
| *TS (45,X) vs. Female (46,XX)* | | | | | | |
| chr2:1413263-1456282 | -1.9732 | -2.423206 | -1.52316 | 5.2737 | 6.8732e-09 | 0.000167 |
| chr12:16376121-16383604 | 2.0676 | 1.740816 | 2.39442 | 5.4364 | 8.4043e-09 | 0.000167 |
| chr1:76313672-76313999 | 1.7856 | 0.945206 | 2.62604 | 5.2963 | 4.1329e-08 | 0.000549 |
| chr16:77319848-77335904 | 1.4621 | 1.173588 | 1.75070 | 4.9756 | 1.2348e-07 | 0.001231 |
| chr3:12305853-12312453 | 1.4843 | 1.136463 | 1.83218 | 5.2864 | 2.7847e-07 | 0.002221 |
| chr6:104428695-104429532 | 1.3906 | 0.410475 | 2.37066 | 4.9472 | 5.3423e-07 | 0.003506 |
| chr6:112185236-112189205 | 1.2536 | 0.868730 | 1.63842 | 4.9277 | 1.0007e-06 | 0.004989 |
| chr6:72295936-72333835 | 2.0401 | 1.716453 | 2.36382 | 6.5771 | 6.1533e-07 | 0.003506 |
| chr5:75411010-75426478 | 1.2283 | 0.821658 | 1.63499 | 4.8202 | 1.2611e-06 | 0.005395 |
| chr21:14825522-14857708 | 1.7335 | 1.350692 | 2.11627 | 5.1215 | 1.3526e-06 | 0.005395 |
| chr2:1137621-1209230 | -1.2970 | -1.759613 | -0.83444 | 4.9964 | 1.8922e-06 | 0.006861 |
| chr7:80656558-80656694 | 1.4040 | 0.931577 | 1.87644 | 4.9097 | 2.2968e-06 | 0.007635 |
| chrX:140783236-140783471 | -1.5452 | -2.049227 | -1.04119 | 5.0011 | 2.5073e-06 | 0.007693 |
| chr6:42637010-42642481 | -1.5149 | -1.930222 | -1.09951 | 4.9985 | 3.0853e-06 | 0.008695 |
| chr8:61633682-61644632 | 1.1802 | 0.806641 | 1.55372 | 4.8919 | 3.2697e-06 | 0.008695 |
| chr6:125045210-125082923 | 1.0801 | -0.095496 | 2.25568 | 4.8474 | 4.8088e-06 | 0.011989 |
| chr4:86772777-86799204 | -1.0358 | -1.435570 | -0.63600 | 4.8567 | 8.6933e-06 | 0.017200 |
| chr11:120431770-120437382 | 1.3105 | 0.974925 | 1.64614 | 4.9453 | 7.9397e-06 | 0.017019 |
| chr1:147252622-147259918 | -1.9317 | -2.354806 | -1.50854 | 5.5583 | 7.7272e-06 | 0.017019 |
| chr13:95985238-95996135 | 1.6433 | 1.287805 | 1.99874 | 5.3849 | 8.1060e-06 | 0.017019 |
| chr5:55343330-55353269 | 1.0280 | 0.592022 | 1.46398 | 4.8123 | 1.1684e-05 | 0.020088 |
| chr7:82949475-82956935 | 1.4218 | 1.069349 | 1.77433 | 5.1436 | 1.2158e-05 | 0.020088 |
| chr2:197459931-197462731 | 1.1691 | 0.845646 | 1.49262 | 4.8799 | 1.6600e-05 | 0.022526 |
| chr9:22820737-22821598 | 1.8346 | 1.464012 | 2.20519 | 6.0388 | 9.0547e-06 | 0.017200 |
| chr11:106939459-106940178 | 1.4935 | 1.137455 | 1.84963 | 5.6365 | 1.2840e-05 | 0.020088 |
| chr1:51075649-51118801 | 1.2792 | 0.848856 | 1.70951 | 4.8331 | 1.6941e-05 | 0.022526 |
| chrX:118614691-118630490 | 1.0346 | 0.612232 | 1.45701 | 4.7404 | 2.1251e-05 | 0.024221 |
| chr5:114404437-114404856 | -1.1319 | -1.464463 | -0.79931 | 6.2192 | 2.0115e-05 | 0.024221 |
| chr15:42634855-42638812 | -1.2588 | -1.605078 | -0.91254 | 5.0716 | 2.0684e-05 | 0.024221 |
| chr2:164743686-164743875 | 1.1521 | 0.711892 | 1.59241 | 4.7839 | 2.6218e-05 | 0.027523 |
| chr9:1327073-1333196 | 1.4732 | 1.117080 | 1.82932 | 5.6313 | 1.7907e-05 | 0.023043 |
| chr5:158941693-158941979 | 2.0736 | 1.600438 | 2.54672 | 5.8502 | 1.5110e-05 | 0.021527 |
| chr1:81836884-81907230 | -1.2806 | -1.761646 | -0.79958 | 5.2089 | 2.8520e-05 | 0.028809 |
| chr7:81965593-81982627 | 1.3045 | 0.258316 | 2.35074 | 5.0332 | 3.2713e-05 | 0.031507 |
| chr5:94888878-94931991 | -1.5827 | -2.119077 | -1.04638 | 5.3003 | 2.8888e-05 | 0.028809 |
| chr8:85281229-85281639 | -1.4395 | -1.787983 | -1.09096 | 4.9787 | 3.6264e-05 | 0.031532 |
| chr12:68851583-68854938 | 1.5672 | 1.242807 | 1.89167 | 4.9451 | 3.3918e-05 | 0.031507 |
| chr1:220761657-220770556 | -1.2539 | -1.682169 | -0.82568 | 5.2759 | 3.8348e-05 | 0.031532 |
| chr3:196368027-196403019 | -1.0463 | -2.206611 | 0.11398 | 4.8192 | 4.0752e-05 | 0.031532 |
| chr19:4508767-4513701 | -1.0450 | -1.434222 | -0.65584 | 4.8280 | 4.1103e-05 | 0.031532 |
| chr12:1141619-1190052 | 1.1858 | 0.841922 | 1.52968 | 4.8667 | 4.2064e-05 | 0.031660 |
| chr10:70152262-70161069 | -1.8072 | -2.809621 | -0.80484 | 5.5238 | 3.7310e-05 | 0.031532 |
| chr2:39331916-39337581 | 1.0696 | 0.723054 | 1.41613 | 5.0174 | 4.8215e-05 | 0.035578 |
| chr11:6941570-6955782 | 1.2063 | 0.831844 | 1.58074 | 5.0968 | 4.9052e-05 | 0.035578 |
| chr18:74630966-74781502 | -1.4170 | -1.737569 | -1.09651 | 5.0856 | 5.2010e-05 | 0.036394 |
| chr5:80775693-80813741 | 1.4655 | 1.077180 | 1.85382 | 5.3567 | 4.0610e-05 | 0.031532 |
| chr12:109239829-109241281 | 1.3227 | 0.956529 | 1.68891 | 5.3253 | 5.0757e-05 | 0.036156 |
| chr12:68851583-68852638 | 1.3419 | 0.920520 | 1.76321 | 4.8525 | 6.7745e-05 | 0.044302 |
| chr12:109191612-109199552 | -1.0149 | -1.344883 | -0.68487 | 4.8868 | 7.8778e-05 | 0.048135 |
| chr12:122288488-122316855 | -1.1593 | -1.522515 | -0.79608 | 4.9607 | 7.9638e-05 | 0.048135 |
| chr16:21315310-21317126 | 1.2572 | 0.903382 | 1.61095 | 5.2192 | 6.6581e-05 | 0.044267 |
| chr6:116689319-116692156 | 1.3878 | 1.047235 | 1.72836 | 5.3389 | 8.5083e-05 | 0.049913 |
| **Muscle** | | | | | | |
| *KS (47,XXY) vs. Male (46,XY)* | | | | | | |
| chr7:128856517-128857336 | -1.3717 | -1.90449 | -0.83887 | 4.8118 | 1.6996e-10 | 6.7802e-06 |
| chr4:67472580-67522512 | 1.0623 | 0.73306 | 1.39161 | 4.7891 | 2.4513e-09 | 4.8894e-05 |
| chr9:35685268-35685551 | 1.3765 | 0.83644 | 1.91647 | 4.8153 | 2.8482e-07 | 2.8405e-03 |
| chr2:178689812-178692099 | -1.5155 | -1.81222 | -1.21875 | 4.8541 | 1.7737e-07 | 2.3585e-03 |
| chr1:56674380-56696159 | -1.0968 | -1.39997 | -0.79372 | 4.7705 | 3.7993e-07 | 3.0312e-03 |
| chr17:10451940-10500752 | -1.5377 | -1.85720 | -1.21820 | 4.9190 | 5.8175e-07 | 3.8678e-03 |
| chr2:95148884-95157439 | 1.3195 | 1.05760 | 1.58147 | 5.0334 | 1.4483e-06 | 6.8256e-03 |
| chr10:68142939-68210547 | 1.0400 | 0.71743 | 1.36249 | 4.9185 | 3.7902e-06 | 1.0800e-02 |
| chr17:10455613-10505511 | 3.5558 | 2.75789 | 4.35367 | 6.0204 | 8.6358e-07 | 4.9214e-03 |
| chr18:27374166-27392867 | 1.2211 | 0.79456 | 1.64755 | 5.0621 | 3.1925e-06 | 9.9594e-03 |
| chr2:160169211-160196420 | 1.1070 | 0.83956 | 1.37441 | 5.0303 | 5.0934e-06 | 1.2699e-02 |
| chr8:74244528-74245175 | -1.3202 | -1.79535 | -0.84510 | 5.3580 | 3.2456e-06 | 9.9594e-03 |
| chr6:123497192-123530565 | -1.1051 | -1.51745 | -0.69275 | 4.9503 | 7.3920e-06 | 1.5520e-02 |
| chr4:15052413-15054217 | 1.0212 | 0.43798 | 1.60433 | 4.9510 | 1.1074e-05 | 2.0081e-02 |
| chr3:195688532-195708841 | -1.9155 | -2.38541 | -1.44568 | 5.2029 | 7.0990e-06 | 1.5520e-02 |
| chr17:10505811-10533632 | -3.1397 | -3.51917 | -2.76022 | 5.1972 | 1.2547e-05 | 2.0211e-02 |
| chr2:151494160-151497033 | -3.2070 | -3.48224 | -2.93166 | 5.7607 | 1.2666e-05 | 2.0211e-02 |
| chr5:68617482-68620047 | 1.3247 | 0.76902 | 1.88036 | 5.0141 | 2.7167e-05 | 3.4960e-02 |
| chr3:125313307-125313656 | -1.1388 | -1.40350 | -0.87400 | 5.0104 | 4.2992e-05 | 4.7235e-02 |
| *TS (45,X) vs. Female (46,XX)* | | | | | | |
| chr5:137880815-137883591 | 2.2552 | 1.77345 | 2.736994 | 5.0945 | 1.0855e-17 | 4.3304e-13 |
| chr2:178650163-178651320 | 3.3613 | 3.00531 | 3.717188 | 5.1049 | 5.8626e-17 | 1.1694e-12 |
| chr10:68142939-68150111 | 2.1904 | 1.80094 | 2.579869 | 5.5504 | 7.0130e-16 | 9.3254e-12 |
| chr4:113249763-113363469 | -2.0338 | -2.48443 | -1.583202 | 4.7852 | 7.5929e-13 | 7.5724e-09 |
| chr7:113882038-113882320 | 2.0312 | 1.63968 | 2.422745 | 5.6595 | 1.3688e-10 | 1.0921e-06 |
| chr11:8413494-8464822 | 2.0100 | 1.51948 | 2.500452 | 5.2907 | 3.3120e-10 | 2.1214e-06 |
| chr2:178681375-178684082 | -3.6772 | -4.16015 | -3.194318 | 5.3712 | 3.7225e-10 | 2.1214e-06 |
| chr1:59900964-59912311 | 1.8342 | 1.41281 | 2.255489 | 5.3281 | 9.6867e-10 | 4.8303e-06 |
| chr17:29079726-29082438 | -1.7220 | -2.08071 | -1.363302 | 4.8356 | 1.4097e-09 | 6.2483e-06 |
| chr12:117225015-117253754 | -1.7174 | -2.31058 | -1.124204 | 4.7549 | 3.5207e-09 | 1.4045e-05 |
| chr9:135850136-135866536 | -2.3601 | -2.79166 | -1.928536 | 5.7768 | 1.0794e-08 | 3.9145e-05 |
| chr7:7506013-7521961 | -1.3247 | -1.70418 | -0.945247 | 4.6954 | 1.6690e-08 | 4.9839e-05 |
| chr2:178681375-178683291 | -1.4346 | -1.86591 | -1.003194 | 4.7122 | 1.8162e-08 | 4.9839e-05 |
| chr1:114732603-114741673 | -1.7537 | -2.18548 | -1.321942 | 4.9200 | 1.7304e-08 | 4.9839e-05 |
| chr13:20788485-20827179 | 1.7983 | 1.07942 | 2.517203 | 5.2256 | 1.8740e-08 | 4.9839e-05 |
| chr12:112075677-112081134 | -1.6384 | -2.06902 | -1.207689 | 4.7878 | 2.3731e-08 | 5.2824e-05 |
| chr17:10505387-10533421 | -2.3617 | -3.06651 | -1.656919 | 4.7733 | 2.3216e-08 | 5.2824e-05 |
| chr1:228291897-228305386 | -1.5102 | -1.84983 | -1.170582 | 4.7262 | 3.0571e-08 | 6.4186e-05 |
| chr10:814741-817889 | 1.5835 | 0.41552 | 2.751439 | 5.2846 | 3.4174e-08 | 6.8163e-05 |
| chr2:178652847-178663710 | 4.2766 | 3.61164 | 4.941572 | 5.4483 | 2.3835e-08 | 5.2824e-05 |
| chr3:184178856-184182248 | 1.3781 | 1.02402 | 1.732253 | 5.0497 | 7.0284e-08 | 1.3351e-04 |
| chr9:109080838-109091152 | 1.2787 | 0.88719 | 1.670304 | 5.0933 | 9.0857e-08 | 1.5102e-04 |
| chr4:73113791-73177533 | -2.0047 | -2.40463 | -1.604676 | 4.8318 | 8.7404e-08 | 1.5102e-04 |
| chr15:25340084-25375763 | -1.6639 | -2.67735 | -0.650480 | 5.0858 | 8.3390e-08 | 1.5102e-04 |
| chr18:3094169-3131496 | -1.7089 | -2.13338 | -1.284322 | 4.8620 | 1.3598e-07 | 2.1698e-04 |
| chr2:178694598-178695380 | -2.0690 | -2.78110 | -1.356920 | 4.9748 | 1.4537e-07 | 2.2304e-04 |
| chr2:197743060-197771982 | -1.6885 | -2.07139 | -1.305534 | 5.0164 | 1.8650e-07 | 2.6931e-04 |
| chr2:189696149-189744788 | 1.6329 | 1.23184 | 2.033872 | 5.2363 | 2.1895e-07 | 2.9770e-04 |
| chr9:105705596-105722621 | 2.0767 | 1.72447 | 2.428855 | 5.6911 | 1.8903e-07 | 2.6931e-04 |
| chr3:27284601-27293652 | -1.4767 | -1.79593 | -1.157453 | 4.7272 | 2.2388e-07 | 2.9770e-04 |
| chr18:21791373-21799974 | -1.2328 | -1.63099 | -0.834601 | 4.7670 | 2.6416e-07 | 3.3993e-04 |
| chr1:219179146-219218712 | 1.3675 | 1.01366 | 1.721292 | 4.9466 | 3.5849e-07 | 4.2061e-04 |
| chr10:37803832-37838111 | 1.5379 | 0.58780 | 2.487918 | 5.4820 | 3.5528e-07 | 4.2061e-04 |
| chr18:63350887-63355299 | -1.4154 | -1.73991 | -1.090893 | 4.7931 | 3.5048e-07 | 4.2061e-04 |
| chr15:42442072-42457158 | -1.3481 | -2.06668 | -0.629468 | 4.7184 | 4.9195e-07 | 5.4513e-04 |
| chr8:42398711-42403461 | 1.6795 | 1.23535 | 2.123737 | 5.4900 | 5.0602e-07 | 5.4557e-04 |
| chr11:22203803-22263043 | 1.5298 | 1.14122 | 1.918323 | 5.5383 | 4.7347e-07 | 5.3965e-04 |
| chr11:94799389-94800311 | 1.7435 | 1.13381 | 2.353215 | 5.8675 | 6.4397e-07 | 6.7527e-04 |
| chr2:24739431-24762776 | -1.5758 | -2.16319 | -0.988354 | 4.9709 | 6.6017e-07 | 6.7527e-04 |
| chr2:178669591-178675995 | -2.7501 | -3.36946 | -2.130691 | 4.8039 | 6.8310e-07 | 6.8125e-04 |
| chr8:97686667-97706750 | -1.4061 | -1.69629 | -1.115957 | 4.7525 | 1.2830e-06 | 1.1902e-03 |
| chr20:35847349-35863400 | -1.8998 | -2.39359 | -1.405995 | 5.2529 | 1.1829e-06 | 1.1235e-03 |
| chr10:101889411-101995355 | -1.1241 | -1.46102 | -0.787243 | 4.7167 | 1.3976e-06 | 1.1935e-03 |
| chr3:119648118-119648668 | 1.6168 | 1.28395 | 1.949595 | 5.4065 | 1.3350e-06 | 1.1935e-03 |
| chr14:85835930-85912234 | -1.2931 | -1.63204 | -0.954246 | 4.7209 | 1.4361e-06 | 1.1935e-03 |
| chr4:109943253-109945275 | -1.3783 | -1.77582 | -0.980726 | 4.8289 | 1.4071e-06 | 1.1935e-03 |
| chr18:20966916-20992937 | 1.7120 | 1.35392 | 2.070046 | 5.8563 | 1.0886e-06 | 1.0592e-03 |
| chr17:7448578-7448788 | -1.5084 | -2.43747 | -0.579234 | 5.2426 | 1.3475e-06 | 1.1935e-03 |
| chr2:178669591-178684082 | 1.8447 | 1.43583 | 2.253641 | 4.9047 | 1.5645e-06 | 1.2589e-03 |
| chr7:44228795-44234676 | -1.1045 | -1.51476 | -0.694179 | 4.6954 | 1.7012e-06 | 1.3226e-03 |
| chr19:19711199-19714487 | -1.5565 | -1.97610 | -1.136825 | 5.0483 | 1.7240e-06 | 1.3226e-03 |
| chr2:178669591-178674409 | 1.3016 | 0.92879 | 1.674477 | 4.8751 | 1.8539e-06 | 1.3954e-03 |
| chr1:77631849-77645967 | -1.3179 | -1.66359 | -0.972257 | 4.6977 | 1.9321e-06 | 1.4126e-03 |
| chr11:106939459-106940178 | 1.3076 | 0.96382 | 1.651452 | 5.6365 | 1.9475e-06 | 1.4126e-03 |
| chr2:178663626-178663902 | -2.8461 | -3.28001 | -2.412247 | 5.0425 | 1.5779e-06 | 1.2589e-03 |
| chr1:26184213-26184507 | -1.0058 | -1.42518 | -0.586384 | 4.7104 | 2.0794e-06 | 1.4517e-03 |
| chr2:55206269-55218600 | -1.4904 | -1.99017 | -0.990561 | 4.9382 | 2.3697e-06 | 1.5907e-03 |
| chr4:76081823-76082535 | 1.5221 | 1.11515 | 1.929107 | 5.6790 | 2.0842e-06 | 1.4517e-03 |
| chr9:94563341-94584669 | -1.4923 | -2.10549 | -0.879152 | 5.2915 | 2.1107e-06 | 1.4517e-03 |
| chr3:49423678-49423959 | -1.5838 | -1.97063 | -1.197043 | 4.7555 | 2.5678e-06 | 1.6793e-03 |
| chr20:21339043-21344208 | 1.8430 | 0.88098 | 2.804958 | 6.3616 | 2.7361e-06 | 1.6883e-03 |
| chr20:20546703-20637501 | -1.4629 | -2.06702 | -0.858735 | 5.0402 | 2.7169e-06 | 1.6883e-03 |
| chr15:84538531-84555070 | -1.4820 | -1.92371 | -1.040344 | 6.5212 | 2.3925e-06 | 1.5907e-03 |
| chr5:151322045-151335547 | 1.2142 | 0.10067 | 2.327644 | 5.1492 | 3.0046e-06 | 1.7890e-03 |
| chr13:100301459-100449305 | -1.5746 | -1.99547 | -1.153782 | 5.3308 | 3.0039e-06 | 1.7890e-03 |
| chr9:124921128-124938866 | -1.3563 | -1.86377 | -0.848770 | 4.7207 | 3.2514e-06 | 1.8726e-03 |
| chr17:10515925-10544127 | 1.3486 | 0.99157 | 1.705571 | 4.9400 | 3.2701e-06 | 1.8726e-03 |
| chr18:63350887-63362868 | -1.4355 | -1.84505 | -1.025866 | 4.9180 | 3.2860e-06 | 1.8726e-03 |
| chr9:33948373-33998864 | -1.1251 | -1.54951 | -0.700659 | 4.7480 | 3.4992e-06 | 1.9200e-03 |
| chr10:24380868-24384423 | 1.5222 | 1.06858 | 1.975872 | 6.9956 | 2.7509e-06 | 1.6883e-03 |
| chr4:113311254-113369805 | 1.2867 | 0.82777 | 1.745716 | 4.9642 | 3.5617e-06 | 1.9200e-03 |
| chr3:37075023-37096660 | 2.0204 | 1.65976 | 2.380952 | 5.4811 | 3.3669e-06 | 1.8917e-03 |
| chr3:81642776-81649917 | -1.1862 | -1.55834 | -0.813967 | 4.7184 | 3.7250e-06 | 1.9553e-03 |
| chr2:151493774-151497033 | -5.0218 | -5.48987 | -4.553677 | 5.4710 | 2.7438e-06 | 1.6883e-03 |
| chr3:119648118-119660063 | -1.3334 | -1.78181 | -0.884913 | 4.9729 | 4.0413e-06 | 2.0669e-03 |
| chr1:46055794-46080750 | -1.8156 | -2.11580 | -1.515397 | 6.2095 | 3.8207e-06 | 1.9794e-03 |
| chr7:81970674-81982627 | 1.5246 | 1.19993 | 1.849307 | 6.5407 | 3.7004e-06 | 1.9553e-03 |
| chr2:175947479-175964589 | -1.1665 | -1.52998 | -0.802969 | 4.7242 | 4.6265e-06 | 2.3362e-03 |
| chr2:151494160-151497033 | 4.3563 | 3.85478 | 4.857766 | 5.7607 | 3.5377e-06 | 1.9200e-03 |
| chr17:69078959-69096801 | -1.2632 | -1.65714 | -0.869312 | 4.7474 | 5.8149e-06 | 2.8996e-03 |
| chr3:175096789-175256530 | -1.1284 | -1.45801 | -0.798783 | 4.8434 | 5.9113e-06 | 2.9113e-03 |
| chr2:135602499-135616757 | -1.1524 | -1.51887 | -0.785929 | 4.7352 | 6.2270e-06 | 2.9739e-03 |
| chr4:25333182-25333988 | 1.4170 | 1.02771 | 1.806223 | 5.9171 | 6.2621e-06 | 2.9739e-03 |
| chr1:66958058-66963160 | 2.0923 | 1.74925 | 2.435334 | 6.5645 | 6.0420e-06 | 2.9393e-03 |
| chr6:87356300-87375710 | -1.9098 | -2.34963 | -1.469983 | 5.0320 | 6.7714e-06 | 3.1779e-03 |
| chr19:38489176-38492636 | 1.1001 | 0.77000 | 1.430143 | 4.9433 | 7.5870e-06 | 3.4789e-03 |
| chr4:109940945-109964537 | -1.4910 | -1.82477 | -1.157137 | 4.9634 | 7.5003e-06 | 3.4789e-03 |
| chr5:79396822-79402055 | -1.0496 | -1.44324 | -0.655899 | 4.7705 | 7.7802e-06 | 3.5269e-03 |
| chr15:71859734-71905006 | -1.1272 | -1.52565 | -0.728700 | 4.7595 | 8.1229e-06 | 3.5499e-03 |
| chr5:37221323-37247745 | -1.3376 | -1.84969 | -0.825581 | 4.8857 | 8.1237e-06 | 3.5499e-03 |
| chr12:110326389-110328017 | -1.4117 | -1.72029 | -1.103094 | 5.1195 | 8.0193e-06 | 3.5499e-03 |
| chr12:21828957-21863054 | -1.1747 | -1.68683 | -0.662650 | 4.7167 | 9.0758e-06 | 3.7586e-03 |
| chr8:61548070-61567318 | -1.1270 | -1.69247 | -0.561492 | 5.2441 | 9.1462e-06 | 3.7586e-03 |
| chr4:140947653-140967873 | -1.3231 | -1.74026 | -0.906009 | 5.1647 | 9.2101e-06 | 3.7586e-03 |
| chr7:32974616-33005466 | -1.1823 | -1.62586 | -0.738679 | 4.8005 | 9.2650e-06 | 3.7586e-03 |
| chr15:75943571-75971248 | -1.0058 | -2.72849 | 0.716842 | 4.7069 | 9.3804e-06 | 3.7586e-03 |
| chr9:109942685-109943381 | -1.2339 | -1.82250 | -0.645300 | 5.4895 | 9.4724e-06 | 3.7586e-03 |
| chr5:140472238-140487060 | -1.6313 | -2.15469 | -1.107828 | 4.9113 | 9.5162e-06 | 3.7586e-03 |
| chr12:22493222-22506819 | 1.7025 | 1.20732 | 2.197613 | 5.3030 | 9.6269e-06 | 3.7651e-03 |
| chr2:178651242-178677287 | 3.2505 | 2.87942 | 3.621556 | 5.4653 | 8.1869e-06 | 3.5499e-03 |
| chr1:243664771-243843282 | -1.1156 | -1.61600 | -0.615251 | 4.7300 | 9.9797e-06 | 3.8348e-03 |
| chr14:31133237-31172122 | -1.5373 | -1.93031 | -1.144300 | 5.1781 | 1.0094e-05 | 3.8348e-03 |
| chr15:66306823-66308844 | -1.3839 | -1.86938 | -0.898367 | 5.1019 | 1.0451e-05 | 3.9332e-03 |
| chr8:140789473-140830526 | -1.4750 | -1.99458 | -0.955336 | 5.4960 | 1.0840e-05 | 4.0413e-03 |
| chr3:183643479-183665034 | -1.1252 | -1.45671 | -0.793770 | 4.7300 | 1.1490e-05 | 4.2131e-03 |
| chr3:152445281-152447773 | 2.2788 | 1.88874 | 2.668857 | 6.6681 | 1.0047e-05 | 3.8348e-03 |
| chr2:151493352-151494253 | 1.3394 | 0.46386 | 2.214979 | 4.9406 | 1.1644e-05 | 4.2131e-03 |
| chr3:184913518-184983094 | 1.1837 | 0.84783 | 1.519492 | 4.9608 | 1.1915e-05 | 4.2131e-03 |
| chr1:99662318-99667766 | -1.5914 | -1.95469 | -1.228160 | 5.4399 | 1.1894e-05 | 4.2131e-03 |
| chr16:19628636-19652090 | -1.4012 | -1.77265 | -1.029756 | 4.9435 | 1.2066e-05 | 4.2221e-03 |
| chr2:178677620-178679416 | 1.7951 | 1.45053 | 2.139752 | 6.3212 | 8.6821e-06 | 3.7242e-03 |
| chr6:145880519-145894977 | -1.1552 | -1.50851 | -0.801793 | 4.8306 | 1.2231e-05 | 4.2428e-03 |
| chr5:75547009-75552591 | -1.9674 | -2.29258 | -1.642191 | 5.9772 | 1.1527e-05 | 4.2131e-03 |
| chr3:172285925-172298787 | -1.1258 | -1.58582 | -0.665801 | 5.0124 | 1.3393e-05 | 4.5676e-03 |
| chr2:108788851-108856970 | -1.1265 | -1.58940 | -0.663698 | 4.7404 | 1.3396e-05 | 4.5676e-03 |
| chr2:11184665-11192712 | 1.2563 | 0.67593 | 1.836667 | 5.1560 | 1.3655e-05 | 4.6161e-03 |
| chr2:159439008-159453801 | -1.2356 | -1.55977 | -0.911392 | 4.7352 | 1.3770e-05 | 4.6161e-03 |
| chr6:123252411-123274670 | 1.3183 | 0.93252 | 1.703982 | 4.8869 | 1.4084e-05 | 4.6820e-03 |
| chr6:75659261-75666941 | -1.1283 | -1.58717 | -0.669504 | 4.8243 | 1.4372e-05 | 4.7384e-03 |
| chr7:129657345-129690546 | -1.7246 | -2.35608 | -1.093203 | 4.9741 | 1.4845e-05 | 4.8542e-03 |
| chr3:81581164-81642990 | -1.4354 | -1.93493 | -0.935841 | 4.9620 | 1.5397e-05 | 4.9935e-03 |
| chr7:131375423-131399433 | 2.1079 | 1.65748 | 2.558346 | 8.2294 | 1.1934e-05 | 4.2131e-03 |
| chr12:122340752-122352786 | -1.4220 | -1.77181 | -1.072212 | 4.8729 | 1.6251e-05 | 5.1863e-03 |
| chr4:94276338-94283303 | -1.2016 | -1.64174 | -0.761533 | 4.7746 | 1.6413e-05 | 5.1965e-03 |
| chr2:39260605-39288280 | -1.1234 | -1.55621 | -0.690556 | 4.7167 | 1.7043e-05 | 5.3115e-03 |
| chr15:87323492-87422036 | -1.3212 | -1.67791 | -0.964505 | 4.9014 | 1.7504e-05 | 5.3913e-03 |
| chr2:97977052-97984536 | -1.1278 | -1.72786 | -0.527795 | 4.7375 | 1.7918e-05 | 5.4564e-03 |
| chr2:178705173-178705320 | -2.3147 | -2.71774 | -1.911753 | 5.1268 | 1.5688e-05 | 5.0471e-03 |
| chrX:109677987-109683372 | -1.1918 | -1.54256 | -0.841120 | 4.7277 | 1.8413e-05 | 5.5646e-03 |
| chr2:178664014-178664537 | -2.4867 | -3.07970 | -1.893740 | 4.8612 | 1.7569e-05 | 5.3913e-03 |
| chr6:111172694-111206735 | -1.6553 | -2.06709 | -1.243535 | 5.0814 | 1.8712e-05 | 5.6125e-03 |
| chr8:22247220-22249309 | -1.2014 | -1.71397 | -0.688813 | 4.7410 | 1.9952e-05 | 5.8957e-03 |
| chr8:94386983-94411315 | -1.6199 | -1.99664 | -1.243069 | 4.9176 | 2.0241e-05 | 5.9371e-03 |
| chr2:178646484-178684082 | -1.3981 | -1.82543 | -0.970855 | 4.8826 | 2.1144e-05 | 6.1567e-03 |
| chr16:25161101-25170805 | -1.5167 | -1.96231 | -1.071180 | 4.9812 | 2.2166e-05 | 6.4077e-03 |
| chr8:2069277-2129232 | -1.1930 | -1.90359 | -0.482372 | 4.7671 | 2.2856e-05 | 6.5594e-03 |
| chr1:228303674-228303938 | -1.4267 | -2.07319 | -0.780300 | 4.8404 | 2.3451e-05 | 6.6821e-03 |
| chr3:122740257-122741113 | 1.3396 | 0.33633 | 2.342918 | 5.3898 | 2.3749e-05 | 6.7192e-03 |
| chr16:28887188-28894953 | -1.2100 | -1.66671 | -0.753196 | 4.7672 | 2.4965e-05 | 6.9643e-03 |
| chr3:134619147-134627818 | 1.0808 | 0.36442 | 1.797188 | 4.9625 | 2.5606e-05 | 7.0936e-03 |
| chr12:67421455-67421784 | -1.2055 | -1.61307 | -0.797847 | 4.7317 | 2.5904e-05 | 7.1265e-03 |
| chr2:226807485-226824800 | -1.0090 | -2.27462 | 0.256602 | 4.6965 | 2.6893e-05 | 7.2657e-03 |
| chr1:151814573-151817280 | -1.2272 | -1.71968 | -0.734644 | 5.0209 | 2.6910e-05 | 7.2657e-03 |
| chr7:156756392-156826744 | 2.0536 | 1.50833 | 2.598787 | 6.6952 | 2.6956e-05 | 7.2657e-03 |
| chr9:105705596-105774026 | 1.1582 | 0.62194 | 1.694488 | 5.0459 | 2.8408e-05 | 7.5567e-03 |
| chr4:151482523-151588926 | -1.2783 | -1.91295 | -0.643685 | 4.9780 | 2.8414e-05 | 7.5567e-03 |
| chr11:47627079-47631087 | 1.3839 | 1.02309 | 1.744649 | 4.9938 | 2.9215e-05 | 7.7182e-03 |
| chr15:63622814-63666472 | -1.3663 | -1.72883 | -1.003816 | 4.7903 | 2.9549e-05 | 7.7550e-03 |
| chr8:65613243-65627433 | 1.2675 | 0.89447 | 1.640431 | 5.0024 | 2.9863e-05 | 7.7863e-03 |
| chr1:92725597-92736627 | -1.6815 | -2.01636 | -1.346714 | 4.9465 | 3.1240e-05 | 8.0513e-03 |
| chr1:117878008-117920424 | -1.1258 | -2.11298 | -0.138614 | 4.7410 | 3.2382e-05 | 8.2806e-03 |
| chr11:77332729-77349287 | -1.2386 | -1.69851 | -0.778649 | 4.8708 | 3.4669e-05 | 8.8091e-03 |
| chr17:34983001-34983552 | -1.3604 | -1.68102 | -1.039755 | 4.9708 | 3.6478e-05 | 9.2100e-03 |
| chr11:22218245-22239684 | -1.2775 | -1.62897 | -0.926066 | 4.9009 | 3.8103e-05 | 9.5000e-03 |
| chr1:84179176-84185182 | -1.0557 | -1.37171 | -0.739790 | 4.7915 | 3.8102e-05 | 9.5000e-03 |
| chr1:84179176-84235364 | -1.2646 | -1.62470 | -0.904529 | 4.9636 | 4.4452e-05 | 1.0879e-02 |
| chr15:58013857-58014281 | -1.0500 | -1.70469 | -0.395391 | 4.8359 | 4.4398e-05 | 1.0879e-02 |
| chr14:39151745-39159550 | -2.0281 | -2.44886 | -1.607346 | 5.7680 | 4.3925e-05 | 1.0879e-02 |
| chr6:89846561-89871388 | -1.7830 | -2.20839 | -1.357683 | 5.0153 | 4.7652e-05 | 1.1521e-02 |
| chr2:178779228-178780034 | -1.2804 | -1.67000 | -0.890776 | 4.7997 | 5.1135e-05 | 1.2142e-02 |
| chr4:109922158-109945275 | -1.5617 | -2.04814 | -1.075304 | 5.4195 | 4.8211e-05 | 1.1586e-02 |
| chr8:67095292-67105975 | -1.2697 | -2.30277 | -0.236652 | 4.8482 | 5.3377e-05 | 1.2430e-02 |
| chr5:88749069-88761125 | -1.2016 | -1.61147 | -0.791753 | 4.7653 | 5.3216e-05 | 1.2430e-02 |
| chr2:178636423-178636560 | -1.2225 | -1.63443 | -0.810501 | 4.7394 | 5.5577e-05 | 1.2742e-02 |
| chr12:9079243-9091429 | -1.0496 | -1.42569 | -0.673432 | 4.8070 | 5.5959e-05 | 1.2756e-02 |
| chr8:19714884-19732566 | -1.2365 | -1.89205 | -0.580924 | 4.8528 | 5.7719e-05 | 1.3083e-02 |
| chrX:32614302-32645152 | -1.0499 | -1.39155 | -0.708305 | 4.7480 | 5.8107e-05 | 1.3096e-02 |
| chr5:7706743-7727261 | -1.3619 | -1.83926 | -0.884479 | 5.0641 | 5.9012e-05 | 1.3225e-02 |
| chr20:2492698-2494044 | -1.1275 | -1.76876 | -0.486145 | 4.8503 | 5.9751e-05 | 1.3316e-02 |
| chr10:70339995-70340702 | -1.2789 | -1.63342 | -0.924411 | 5.2467 | 6.0354e-05 | 1.3376e-02 |
| chr18:8244057-8253414 | 1.1259 | 0.78680 | 1.464984 | 5.7488 | 6.4431e-05 | 1.4200e-02 |
| chr1:111456085-111459636 | 1.1559 | 0.76131 | 1.550583 | 5.3535 | 6.5931e-05 | 1.4451e-02 |
| chr6:73610399-73644603 | -1.1275 | -1.44652 | -0.808490 | 4.8418 | 7.2385e-05 | 1.5693e-02 |
| chr13:20002849-20005239 | -1.1280 | -1.50400 | -0.752054 | 4.7745 | 7.3310e-05 | 1.5743e-02 |
| chr2:54526371-54526566 | -1.1821 | -1.54685 | -0.817360 | 5.3369 | 7.5333e-05 | 1.5985e-02 |
| chr6:123331878-123352586 | -1.5002 | -2.10212 | -0.898207 | 4.8033 | 7.6035e-05 | 1.6049e-02 |
| chr1:225507950-225514900 | -1.0503 | -1.44052 | -0.660178 | 4.8828 | 7.4826e-05 | 1.5962e-02 |
| chr2:189346224-189348196 | -1.0512 | -1.44600 | -0.656314 | 5.0253 | 7.8379e-05 | 1.6456e-02 |
| chr18:8714138-8720496 | -1.0917 | -1.47811 | -0.705307 | 5.0211 | 8.2509e-05 | 1.7143e-02 |
| chr1:117460488-117522937 | -1.3632 | -1.77896 | -0.947516 | 5.1736 | 8.6309e-05 | 1.7657e-02 |
| chr7:116699070-116700284 | 1.1945 | 0.63123 | 1.757718 | 6.1667 | 8.6309e-05 | 1.7657e-02 |
| chr17:62536169-62565137 | -1.1283 | -1.52227 | -0.734333 | 4.7930 | 8.5399e-05 | 1.7651e-02 |
| chr19:52373110-52406643 | -1.2333 | -1.70467 | -0.762012 | 4.9616 | 8.9791e-05 | 1.8091e-02 |
| chr7:148753999-148767749 | -1.0152 | -1.57998 | -0.450457 | 4.7433 | 8.9602e-05 | 1.8091e-02 |
| chr20:13766776-13776270 | -1.2886 | -1.66498 | -0.912220 | 4.9192 | 9.3296e-05 | 1.8351e-02 |
| chr14:37285317-37308519 | 1.3902 | 1.05469 | 1.725691 | 5.7366 | 9.3386e-05 | 1.8351e-02 |
| chr2:151565720-151567382 | -1.0720 | -1.56893 | -0.575038 | 4.7181 | 9.2392e-05 | 1.8337e-02 |
| chr14:70223561-70227704 | -1.2601 | -2.02620 | -0.494048 | 5.2815 | 9.1222e-05 | 1.8250e-02 |
| chr13:39694633-39751144 | 1.1550 | 0.73945 | 1.570529 | 5.0832 | 9.4693e-05 | 1.8517e-02 |
| chr5:80434108-80440032 | -1.2006 | -1.54816 | -0.853027 | 4.7294 | 9.7592e-05 | 1.8717e-02 |
| chr10:29550596-29569312 | 1.6680 | 1.14540 | 2.190572 | 5.5611 | 9.8767e-05 | 1.8852e-02 |
| chr1:227028656-227035607 | -1.2040 | -1.52663 | -0.881438 | 5.0455 | 9.9834e-05 | 1.8875e-02 |
| chr3:131132744-131133956 | 1.1147 | 0.68652 | 1.542825 | 5.3397 | 1.0298e-04 | 1.9287e-02 |
| chr15:70664661-70669462 | -1.0947 | -1.48641 | -0.703057 | 4.7798 | 1.0627e-04 | 1.9809e-02 |
| chr4:113609151-113679496 | -1.4665 | -1.83409 | -1.098945 | 5.2002 | 1.0777e-04 | 1.9997e-02 |
| chr8:42906172-42982696 | -1.1024 | -1.60794 | -0.596933 | 4.7219 | 1.1014e-04 | 2.0247e-02 |
| chr2:178662965-178664926 | 1.1475 | 0.58183 | 1.713202 | 5.2645 | 1.1455e-04 | 2.0677e-02 |
| chr17:66053735-66070001 | -1.1290 | -1.58402 | -0.673922 | 5.1368 | 1.2292e-04 | 2.1891e-02 |
| chr10:27067156-27092512 | -1.0980 | -1.48031 | -0.715697 | 4.9017 | 1.2527e-04 | 2.2112e-02 |
| chr7:64521063-64547382 | -1.0441 | -1.43546 | -0.652724 | 4.7254 | 1.2595e-04 | 2.2134e-02 |
| chr2:178685517-178688224 | 1.2219 | 0.85967 | 1.584134 | 4.9441 | 1.2832e-04 | 2.2290e-02 |
| chr1:59346246-59378837 | 1.1923 | 0.72533 | 1.659196 | 5.1802 | 1.2852e-04 | 2.2290e-02 |
| chr6:56610426-56614484 | 1.0628 | 0.73750 | 1.388021 | 5.0474 | 1.2850e-04 | 2.2290e-02 |
| chr1:94455732-94464873 | -1.0424 | -2.01392 | -0.070838 | 4.8011 | 1.3720e-04 | 2.3592e-02 |
| chrX:338603-347693 | -2.0072 | -2.41766 | -1.596713 | 5.4313 | 1.4818e-04 | 2.5153e-02 |
| chr1:235465635-235494828 | -1.1388 | -1.75026 | -0.527297 | 4.8203 | 1.5237e-04 | 2.5647e-02 |
| chr2:151690726-151697648 | -1.0332 | -1.46622 | -0.600161 | 4.7966 | 1.5478e-04 | 2.5835e-02 |
| chr4:39872985-39890364 | -1.3193 | -1.79494 | -0.843576 | 4.9585 | 1.5451e-04 | 2.5835e-02 |
| chr5:88804597-88823927 | -1.4797 | -1.85380 | -1.105592 | 5.0198 | 1.6079e-04 | 2.6596e-02 |
| chr4:113363337-113374946 | -1.0531 | -1.67030 | -0.435805 | 4.8329 | 1.5742e-04 | 2.6166e-02 |
| chr13:77126317-77129230 | 1.6103 | 1.19461 | 2.026069 | 5.7173 | 1.6472e-04 | 2.6988e-02 |
| chr3:134608648-134625135 | -1.1861 | -1.74583 | -0.626377 | 4.8757 | 1.6527e-04 | 2.6988e-02 |
| chr2:178679338-178683291 | 1.1722 | 0.64208 | 1.702250 | 5.1297 | 1.6922e-04 | 2.7330e-02 |
| chr2:178689812-178693689 | 1.1930 | 0.48961 | 1.896395 | 5.3506 | 1.7449e-04 | 2.8067e-02 |
| chr2:29133654-29157402 | -1.2777 | -1.71419 | -0.841172 | 5.3798 | 1.7903e-04 | 2.8610e-02 |
| chr1:235465635-235484515 | -1.3593 | -1.77183 | -0.946812 | 5.0847 | 1.8386e-04 | 2.8990e-02 |
| chr14:23423906-23424092 | -1.0672 | -1.38549 | -0.748821 | 4.7185 | 1.7930e-04 | 2.8610e-02 |
| chr9:106972004-106974273 | -1.2527 | -1.73002 | -0.775363 | 5.1036 | 1.8645e-04 | 2.9283e-02 |
| chr1:43920403-43921638 | 1.6769 | 1.01711 | 2.336638 | 5.5838 | 1.8764e-04 | 2.9312e-02 |
| chr10:91951402-91953873 | -1.0523 | -1.40989 | -0.694621 | 4.9239 | 1.8810e-04 | 2.9312e-02 |
| chr11:103219914-103223086 | -1.0343 | -1.75072 | -0.317795 | 5.3256 | 1.9255e-04 | 2.9887e-02 |
| chr2:152132113-152150229 | 1.4305 | 1.05571 | 1.805369 | 5.2011 | 1.9440e-04 | 2.9978e-02 |
| chr16:2756333-2757945 | -1.1260 | -1.92431 | -0.327713 | 4.7410 | 1.9566e-04 | 2.9989e-02 |
| chr7:77273693-77280653 | -1.0472 | -1.46707 | -0.627339 | 4.8990 | 1.9463e-04 | 2.9978e-02 |
| chr12:99772921-99806700 | -1.0469 | -1.45481 | -0.638922 | 5.0786 | 1.9786e-04 | 3.0125e-02 |
| chr6:123464905-123571132 | 1.4238 | 1.10131 | 1.746364 | 5.5515 | 2.0293e-04 | 3.0201e-02 |
| chr17:10497733-10525616 | -2.2442 | -2.59039 | -1.897939 | 4.9133 | 2.0517e-04 | 3.0201e-02 |
| chr7:113878135-113882320 | -1.2555 | -1.77367 | -0.737258 | 4.9647 | 2.0839e-04 | 3.0220e-02 |
| chr2:101059420-101068503 | -1.0946 | -1.43061 | -0.758511 | 4.8097 | 2.0761e-04 | 3.0220e-02 |
| chr12:31442774-31495919 | -1.4900 | -2.01943 | -0.960593 | 5.1072 | 2.0984e-04 | 3.0220e-02 |
| chr10:96155325-96160402 | 1.6490 | 1.22043 | 2.077543 | 5.8883 | 2.1106e-04 | 3.0286e-02 |
| chr21:46484756-46511614 | -1.6883 | -2.13394 | -1.242666 | 4.9260 | 2.1946e-04 | 3.1156e-02 |
| chr9:109087172-109091155 | 1.1288 | 0.58697 | 1.670705 | 5.3131 | 2.2390e-04 | 3.1673e-02 |
| chr3:138570317-138571356 | 2.4652 | 1.96882 | 2.961551 | 6.2935 | 2.2609e-04 | 3.1680e-02 |
| chr9:77219953-77228264 | -1.0603 | -1.50630 | -0.614277 | 4.7590 | 2.1786e-04 | 3.1039e-02 |
| chr10:27063987-27079161 | -1.1845 | -1.59305 | -0.775942 | 4.8069 | 2.2712e-04 | 3.1680e-02 |
| chr4:142260491-142270732 | -1.3075 | -1.84198 | -0.773048 | 4.7665 | 2.3448e-04 | 3.2554e-02 |
| chr11:66369059-66369532 | -1.2144 | -1.57752 | -0.851326 | 4.9504 | 2.4416e-04 | 3.3357e-02 |
| chr1:228299881-228300145 | 1.1322 | 0.57205 | 1.692400 | 4.8669 | 2.4886e-04 | 3.3430e-02 |
| chr3:175096789-175097291 | -1.2543 | -1.58975 | -0.918907 | 5.1161 | 2.4852e-04 | 3.3430e-02 |
| chr2:151498259-151502885 | -1.2538 | -1.74931 | -0.758222 | 4.9496 | 2.5470e-04 | 3.3921e-02 |
| chr5:38496381-38530666 | -1.4348 | -2.09685 | -0.772682 | 5.5358 | 2.6016e-04 | 3.3956e-02 |
| chr2:178695864-178696041 | -1.9518 | -2.44313 | -1.460377 | 5.0623 | 2.5865e-04 | 3.3956e-02 |
| chr2:17695151-17745951 | -1.8521 | -2.20480 | -1.499476 | 4.9784 | 2.6608e-04 | 3.4240e-02 |
| chr8:86442618-86452679 | 1.0197 | 0.65011 | 1.389331 | 4.9451 | 2.5918e-04 | 3.3956e-02 |
| chr2:33527136-33534400 | -1.0028 | -1.41179 | -0.593864 | 4.7560 | 2.5921e-04 | 3.3956e-02 |
| chr6:170543600-170549113 | 1.6933 | 1.24083 | 2.145672 | 6.6089 | 2.7495e-04 | 3.5155e-02 |
| chr17:39297126-39303639 | -1.1318 | -1.65477 | -0.608811 | 4.8596 | 2.7239e-04 | 3.4940e-02 |
| chr2:178679598-178684082 | -1.0556 | -1.41406 | -0.697126 | 4.7508 | 2.7783e-04 | 3.5184e-02 |
| chr12:124419956-124457162 | -1.4568 | -1.84776 | -1.065746 | 5.5997 | 3.0362e-04 | 3.6372e-02 |
| chr4:76996424-77030970 | -1.0689 | -1.41055 | -0.727318 | 5.7675 | 2.9419e-04 | 3.6372e-02 |
| chr7:39987598-40047877 | -1.0538 | -1.47010 | -0.637514 | 5.0151 | 2.9507e-04 | 3.6372e-02 |
| chr2:56560153-56593673 | -1.0709 | -1.69271 | -0.449010 | 4.7759 | 2.9987e-04 | 3.6372e-02 |
| chr3:171188701-171228221 | -1.0196 | -1.31825 | -0.720948 | 4.7462 | 3.1678e-04 | 3.7059e-02 |
| chr7:38010675-38013581 | -1.7426 | -2.43264 | -1.052470 | 5.0912 | 3.2395e-04 | 3.7315e-02 |
| chr2:178664014-178664737 | 1.5020 | 1.04008 | 1.963943 | 4.9257 | 3.2124e-04 | 3.7252e-02 |
| chr7:23516362-23522432 | -1.2014 | -1.61010 | -0.792690 | 4.7728 | 3.1563e-04 | 3.7032e-02 |
| chr16:72958599-72960194 | -1.0767 | -1.45496 | -0.698508 | 4.7711 | 3.1986e-04 | 3.7252e-02 |
| chr2:206866696-206881891 | 1.6043 | 1.08172 | 2.126786 | 5.5621 | 3.2774e-04 | 3.7450e-02 |
| chr1:243270104-243308177 | -1.0686 | -1.44778 | -0.689412 | 4.9674 | 3.2309e-04 | 3.7315e-02 |
| chr2:210040419-210075756 | -1.2796 | -1.68172 | -0.877484 | 4.9176 | 3.3574e-04 | 3.7941e-02 |
| chr1:154172907-154176248 | 1.1857 | 0.66826 | 1.703226 | 4.9610 | 3.2459e-04 | 3.7315e-02 |
| chr3:195959011-195960086 | -1.9575 | -2.27661 | -1.638339 | 5.7865 | 3.4571e-04 | 3.8489e-02 |
| chr2:151570080-151603772 | 1.0964 | 0.70050 | 1.492377 | 4.8417 | 3.4454e-04 | 3.8489e-02 |
| chr15:100132006-100155320 | -1.2371 | -1.79269 | -0.681554 | 4.7427 | 3.6146e-04 | 3.9833e-02 |
| chr6:124931695-124948098 | -1.4977 | -1.84954 | -1.145893 | 5.4027 | 3.6632e-04 | 4.0256e-02 |
| chr4:169507036-169508852 | 1.0144 | 0.58778 | 1.440993 | 5.0754 | 3.6849e-04 | 4.0384e-02 |
| chr1:52465069-52490801 | -1.1806 | -1.57525 | -0.785906 | 4.8416 | 3.8328e-04 | 4.1210e-02 |
| chr8:67045811-67059445 | -1.1179 | -1.57390 | -0.661900 | 5.0299 | 3.9417e-04 | 4.1590e-02 |
| chr14:92126121-92143294 | 1.7301 | 1.19551 | 2.264678 | 6.7277 | 3.9625e-04 | 4.1590e-02 |
| chrX:32565701-32573846 | -1.3649 | -1.86452 | -0.865339 | 5.3407 | 3.9826e-04 | 4.1590e-02 |
| chr13:42168439-42210765 | -1.1721 | -1.54980 | -0.794473 | 4.8011 | 4.0680e-04 | 4.2151e-02 |
| chr2:23968918-23984831 | -1.1610 | -1.51543 | -0.806539 | 4.7850 | 3.9611e-04 | 4.1590e-02 |
| chr7:72713620-72744601 | -1.3323 | -1.87097 | -0.793599 | 5.0441 | 4.0630e-04 | 4.2151e-02 |
| chr2:178753123-178786141 | -1.0272 | -1.43765 | -0.616797 | 4.7291 | 4.0814e-04 | 4.2156e-02 |
| chr17:66027500-66070001 | -1.0807 | -1.42928 | -0.732216 | 5.1320 | 4.1720e-04 | 4.2637e-02 |
| chr3:86968589-86969123 | -1.0425 | -1.69975 | -0.385329 | 5.5984 | 4.1068e-04 | 4.2156e-02 |
| chr6:56631210-56636652 | -1.0375 | -1.46620 | -0.608743 | 4.9490 | 4.2230e-04 | 4.2866e-02 |
| chr17:10505811-10533632 | -3.4747 | -3.98233 | -2.967038 | 5.1972 | 4.3312e-04 | 4.3853e-02 |
| chr3:174519076-174550637 | 1.0148 | 0.61899 | 1.410514 | 4.9780 | 4.3747e-04 | 4.4069e-02 |
| chr20:32366383-32369123 | 1.9590 | 1.53192 | 2.386152 | 7.7507 | 4.5333e-04 | 4.4648e-02 |
| chr12:56159586-56160320 | 1.2551 | 0.62518 | 1.884921 | 5.5829 | 4.6184e-04 | 4.5045e-02 |
| chr9:105720734-105774026 | 1.0582 | 0.61357 | 1.502737 | 5.0628 | 4.5245e-04 | 4.4648e-02 |
| chr3:23589733-23595250 | -1.0740 | -2.03021 | -0.117766 | 4.9885 | 4.3555e-04 | 4.3987e-02 |
| chr1:66940027-66963160 | -1.3541 | -1.80703 | -0.901263 | 4.9770 | 4.5440e-04 | 4.4648e-02 |
| chr1:20893516-20942330 | -1.8751 | -2.23217 | -1.518000 | 5.2344 | 4.7394e-04 | 4.5448e-02 |
| chr2:169482250-169499620 | -1.1126 | -1.44022 | -0.784920 | 4.8734 | 4.6783e-04 | 4.5298e-02 |
| chr15:50637421-50648885 | -1.1231 | -2.10823 | -0.137968 | 4.8944 | 4.5988e-04 | 4.5045e-02 |
| chr15:29761138-29800702 | -1.2135 | -1.93482 | -0.492276 | 5.6524 | 4.9460e-04 | 4.6425e-02 |
| chr10:93670769-93687449 | -1.3048 | -1.66268 | -0.946962 | 5.2583 | 4.8496e-04 | 4.5844e-02 |
| chr18:3164277-3215251 | -1.0653 | -1.42451 | -0.706070 | 4.8589 | 4.8031e-04 | 4.5729e-02 |
| chr14:92809299-92816421 | -1.0459 | -1.43297 | -0.658817 | 4.9064 | 4.7324e-04 | 4.5448e-02 |
| chr5:34020586-34052110 | -1.1462 | -1.61832 | -0.674052 | 4.8682 | 4.9437e-04 | 4.6425e-02 |
| chr2:61293463-61350693 | -1.0893 | -1.39876 | -0.779937 | 4.8913 | 5.1351e-04 | 4.6789e-02 |
| chr10:118729294-118730410 | -1.4005 | -1.85463 | -0.946420 | 5.2719 | 5.4808e-04 | 4.8160e-02 |
| chr8:22498000-22498489 | -1.2365 | -1.74901 | -0.724012 | 4.9216 | 5.4022e-04 | 4.8021e-02 |
| chr12:123484179-123499536 | -1.1836 | -2.00868 | -0.358607 | 4.8549 | 5.6459e-04 | 4.9095e-02 |
| chr19:47143493-47155213 | -1.1285 | -1.48128 | -0.775777 | 4.8289 | 5.3866e-04 | 4.8021e-02 |
| chr16:81354404-81365478 | -1.2904 | -1.88726 | -0.693618 | 5.1019 | 5.8040e-04 | 4.9840e-02 |
| chr2:151630714-151636334 | -1.2315 | -1.64892 | -0.814147 | 4.8377 | 5.8327e-04 | 4.9840e-02 |
| chr18:9931809-9950568 | 1.7560 | 1.33324 | 2.178783 | 6.6310 | 5.8967e-04 | 4.9928e-02 |
| chrX:112804946-112815877 | -1.5660 | -2.22400 | -0.907935 | 5.7434 | 5.8102e-04 | 4.9840e-02 |
| chr6:170537233-170549113 | 1.3718 | 0.95667 | 1.786878 | 5.9014 | 5.8398e-04 | 4.9840e-02 |
| chr13:100307191-100340259 | -1.0842 | -1.55927 | -0.609148 | 5.5034 | 5.9075e-04 | 4.9928e-02 |
| chr2:169946287-169958526 | -1.0442 | -1.40285 | -0.685612 | 4.9174 | 5.7365e-04 | 4.9748e-02 |

logFC; log2 fold change, CI.L; confidence interval left, CI.R; confidence interval right, AveExpr; average expression, adj.P.Val;

**Supplementary Table 2. Differential expression analysis of genes from the pseudoautosomal regions.**

| Gene | logFC | CI.L | CI.R | AveExpr | P.Value | adj.P.Val |
| --- | --- | --- | --- | --- | --- | --- |
| **Blood** | | | | | | |
| *KS (47,XXY) vs. Male (46,XY)* | | | | | | |
| AKAP17A | 0.44922 | 0.344681 | 0.553758 | 4.48643 | 5.1373e-15 | 1.9340e-11 |
| ASMTL | 0.38642 | 0.240189 | 0.532652 | 3.55780 | 4.6623e-07 | 4.2549e-04 |
| CD99 | 0.39555 | 0.238272 | 0.552836 | 6.25175 | 1.5075e-06 | 1.0806e-03 |
| CSF2RA | 0.22133 | -0.040209 | 0.482875 | 3.46302 | 9.6734e-02 | 4.5259e-01 |
| DHRSX | 0.35214 | 0.182692 | 0.521585 | 3.66752 | 6.0546e-05 | 1.4946e-02 |
| GTPBP6 | 0.49729 | 0.353115 | 0.641460 | 4.28610 | 1.1693e-10 | 2.9130e-07 |
| IL3RA | 0.15964 | -0.228204 | 0.547475 | 2.12508 | 4.1797e-01 | 6.4738e-01 |
| IL9R | 0.33560 | -0.239115 | 0.910319 | -2.65333 | 2.5091e-01 | 5.2996e-01 |
| P2RY8 | 0.41084 | 0.291626 | 0.530054 | 3.90159 | 1.2090e-10 | 2.9130e-07 |
| PLCXD1 | 0.57548 | 0.301435 | 0.849530 | 2.95161 | 5.0997e-05 | 1.3127e-02 |
| PPP2R3B | 0.61472 | 0.453573 | 0.775875 | 3.21118 | 1.7857e-12 | 5.3781e-09 |
| SHOX | 0.15648 | -0.730829 | 1.043783 | -3.08724 | 7.2840e-01 | 8.4837e-01 |
| SLC25A6 | 0.52221 | 0.381242 | 0.663174 | 6.58183 | 6.4522e-12 | 1.7665e-08 |
| SPRY3 | -0.12804 | -0.287750 | 0.031663 | 2.43465 | 1.1547e-01 | 4.5259e-01 |
| VAMP7 | -0.21464 | -0.366577 | -0.062710 | 4.23253 | 5.8533e-03 | 1.7857e-01 |
| XG | 0.71828 | 0.032454 | 1.404097 | -0.46396 | 4.0193e-02 | 4.3574e-01 |
| ZBED1 | 0.30758 | 0.174554 | 0.440601 | 5.72209 | 8.9238e-06 | 4.0720e-03 |
| *TS (45,X) vs. Female (46,XX)* | | | | | | |
| AKAP17A | -0.86669034 | -0.95651 | -0.776874 | 4.48643 | 9.0880e-47 | 1.0948e-42 |
| ASMTL | -0.87819996 | -1.00515 | -0.751249 | 3.55780 | 2.1789e-30 | 9.3744e-27 |
| CD99 | -0.83586580 | -0.98093 | -0.690806 | 6.25175 | 2.0760e-23 | 4.4658e-20 |
| CSF2RA | -0.48541803 | -0.70738 | -0.263453 | 3.46302 | 2.5346e-05 | 9.8042e-04 |
| DHRSX | -0.42395305 | -0.56485 | -0.283055 | 3.66752 | 1.2883e-08 | 2.1859e-06 |
| GTPBP6 | -1.01587645 | -1.14171 | -0.890048 | 4.28610 | 2.0809e-37 | 1.3927e-33 |
| IL3RA | -1.02017970 | -1.35280 | -0.687560 | 2.12508 | 7.0846e-09 | 1.3542e-06 |
| IL9R | -0.52119058 | -0.98665 | -0.055734 | -2.65333 | 2.8380e-02 | 1.8125e-01 |
| P2RY8 | -0.92727572 | -1.03294 | -0.821608 | 3.90159 | 1.2788e-41 | 9.6285e-38 |
| PLCXD1 | -0.99672197 | -1.23358 | -0.759866 | 2.95161 | 1.5502e-14 | 1.3936e-11 |
| PPP2R3B | -0.97177830 | -1.11170 | -0.831856 | 3.21118 | 1.4818e-30 | 6.8659e-27 |
| SHOX | -0.00023248 | -0.70305 | 0.702588 | -3.08724 | 9.9948e-01 | 9.9976e-01 |
| SLC25A6 | -0.97448190 | -1.10353 | -0.845431 | 6.58183 | 3.0435e-34 | 1.8332e-30 |
| SPRY3 | 0.39976293 | 0.28153 | 0.517994 | 2.43465 | 2.4811e-10 | 8.1458e-08 |
| VAMP7 | 0.27765375 | 0.15867 | 0.396640 | 4.23253 | 7.4411e-06 | 3.6262e-04 |
| XG | -1.18882114 | -1.71080 | -0.666838 | -0.46396 | 1.1966e-05 | 5.3075e-04 |
| ZBED1 | -0.48435643 | -0.60032 | -0.368390 | 5.72209 | 2.2836e-14 | 1.9934e-11 |
| **Fat** | | | | | | |
| *KS (47,XXY) vs. Male (46,XY)* | | | | | | |
| AKAP17A | 0.734893 | 0.582591 | 0.887194 | 4.48643 | 6.0412e-18 | 9.0969e-14 |
| ASMTL | 0.369051 | 0.224248 | 0.513854 | 3.55780 | 1.1100e-06 | 1.2615e-03 |
| CD99 | 0.491781 | 0.367911 | 0.615651 | 6.25175 | 2.8121e-13 | 1.8820e-09 |
| CSF2RA | 0.774071 | 0.367077 | 1.181066 | 3.46302 | 2.3114e-04 | 3.9656e-02 |
| DHRSX | 0.407653 | 0.210364 | 0.604942 | 3.66752 | 6.6463e-05 | 1.9741e-02 |
| GTPBP6 | 0.520253 | 0.371788 | 0.668718 | 4.28610 | 6.3288e-11 | 2.9323e-07 |
| IL3RA | 0.275065 | -0.093967 | 0.644098 | 2.12508 | 1.4319e-01 | 6.3778e-01 |
| IL9R | 1.089467 | 0.181440 | 1.997493 | -2.65333 | 1.8941e-02 | 3.5534e-01 |
| P2RY8 | 0.112307 | -0.126367 | 0.350981 | 3.90159 | 3.5460e-01 | 6.8159e-01 |
| PLCXD1 | 0.437724 | 0.162212 | 0.713236 | 2.95161 | 1.9908e-03 | 1.2759e-01 |
| PPP2R3B | 0.716969 | 0.508597 | 0.925341 | 3.21118 | 1.2843e-10 | 5.5257e-07 |
| SHOX | 0.833269 | -0.117300 | 1.783837 | -3.08724 | 8.5430e-02 | 6.3778e-01 |
| SLC25A6 | 0.372547 | 0.245321 | 0.499773 | 6.58183 | 2.9168e-08 | 7.9858e-05 |
| SPRY3 | -0.028816 | -0.202509 | 0.144876 | 2.43465 | 7.4390e-01 | 8.7753e-01 |
| VAMP7 | -0.048780 | -0.193567 | 0.096007 | 4.23253 | 5.0723e-01 | 7.6118e-01 |
| XG | 0.214092 | -0.169661 | 0.597845 | -0.46396 | 2.7261e-01 | 6.3778e-01 |
| ZBED1 | 0.326109 | 0.187997 | 0.464221 | 5.72209 | 5.8649e-06 | 4.2561e-03 |
| *TS (45,X) vs. Female (46,XX)* | | | | | | |
| AKAP17A | -0.74147 | -0.921635 | -0.56131 | 4.48643 | 4,83E-10 | 3,64E-06 |
| ASMTL | -0.83231 | -1.028.880 | -0.63575 | 3.55780 | 1,12E-10 | 1,13E-06 |
| CD99 | -0.75978 | -0.940348 | -0.57921 | 6.25175 | 1,56E-10 | 1,34E-06 |
| CSF2RA | -0.21700 | -0.709896 | 0.27590 | 3.46302 | 3,86E+03 | 9,60E+03 |
| DHRSX | -0.58817 | -0.852087 | -0.32424 | 3.66752 | 1,80E-01 | 2,24E+01 |
| GTPBP6 | -0.75944 | -0.950129 | -0.56874 | 4.28610 | 2,42E-09 | 1,62E-05 |
| IL3RA | -100.175 | -1.510.167 | -0.49333 | 2.12508 | 1,39E+00 | 8,40E+01 |
| IL9R | -0.56320 | -1.654.037 | 0.52763 | -265.333 | 3,10E+03 | 9,08E+03 |
| P2RY8 | -0.79648 | -1.084.249 | -0.50872 | 3.90159 | 1,42E-03 | 7,62E-01 |
| PLCXD1 | -0.19027 | -0.566543 | 0.18601 | 2.95161 | 3,20E+03 | 9,21E+03 |
| PPP2R3B | -0.88817 | -1.134.170 | -0.64218 | 3.21118 | 1,90E-07 | 6,02E-04 |
| SHOX | -0.42607 | -1.715.733 | 0.86360 | -308.724 | 5,16E+03 | 9,60E+03 |
| SLC25A6 | -0.89387 | -1.075.130 | -0.71261 | 6.58183 | 1,50E-14 | 3,02E-10 |
| SPRY3 | 0.17431 | -0.037026 | 0.38564 | 2.43465 | 1,05E+03 | 5,23E+03 |
| VAMP7 | 0.32442 | 0.139581 | 0.50925 | 4.23253 | 6,58E+00 | 2,26E+02 |
| XG | -0.36372 | -0.900610 | 0.17316 | -0.46396 | 1,83E+03 | 6,96E+03 |
| ZBED1 | -0.65142 | -0.833471 | -0.46937 | 5.72209 | 2,74E-07 | 7,51E-04 |
| **Muscle** | | | | | | |
| *KS (47,XXY) vs. Male (46,XY)* | | | | | | |
| AKAP17A | 0.5367104 | 0.385491 | 0.68793 | 4.48643 | 3.8128e-11 | 1.2759e-07 |
| ASMTL | 0.2071253 | 0.050677 | 0.36357 | 3.55780 | 9.7218e-03 | 2.2205e-01 |
| CD99 | 0.3772093 | 0.211534 | 0.54288 | 6.25175 | 1.2038e-05 | 4.7390e-03 |
| CSF2RA | 0.6554521 | -0.197836 | 1.50874 | 3.46302 | 1.3143e-01 | 3.6520e-01 |
| DHRSX | 0.5079939 | 0.280800 | 0.73519 | 3.66752 | 1.6932e-05 | 5.9595e-03 |
| GTPBP6 | 0.3886376 | 0.261823 | 0.51545 | 4.28610 | 7.2692e-09 | 1.2878e-05 |
| IL3RA | 0.4319630 | -0.018803 | 0.88273 | 2.12508 | 6.0253e-02 | 2.2923e-01 |
| IL9R | 0.2075046 | -0.593868 | 1.00888 | -2.65333 | 6.1020e-01 | 7.8595e-01 |
| P2RY8 | 0.5144294 | 0.245100 | 0.78376 | 3.90159 | 2.1774e-04 | 2.8149e-02 |
| PLCXD1 | 0.4484309 | 0.027975 | 0.86889 | 2.95161 | 3.6708e-02 | 2.2205e-01 |
| PPP2R3B | 0.4331851 | 0.328933 | 0.53744 | 3.21118 | 2.9760e-14 | 1.4938e-10 |
| SHOX | -0.4563507 | -1.420680 | 0.50798 | -3.08724 | 3.5186e-01 | 6.0683e-01 |
| SLC25A6 | 0.4615366 | 0.272740 | 0.65033 | 6.58183 | 2.8276e-06 | 1.8513e-03 |
| SPRY3 | 0.1491006 | -0.035541 | 0.33374 | 2.43465 | 1.1289e-01 | 3.3507e-01 |
| VAMP7 | 0.0015729 | -0.159756 | 0.16290 | 4.23253 | 9.8468e-01 | 9.9276e-01 |
| XG | 0.1601199 | -0.364426 | 0.68467 | -0.46396 | 5.4790e-01 | 7.4334e-01 |
| ZBED1 | 0.2798139 | 0.168578 | 0.39105 | 5.72209 | 1.4997e-06 | 1.1016e-03 |
| *TS (45,X) vs. Female (46,XX)* | | | | | | |
| AKAP17A | -0.7851 | -1.0443 | -0.5258 | 4.486 | 1.056e-08 | 2.891e-05 |
| ASMTL | -0.4839 | -0.7675 | -0.2002 | 3.558 | 9.200e-04 | 9.114e-02 |
| CD99 | -0.6895 | -1.0220 | -0.3570 | 6.252 | 6.275e-05 | 1.680e-02 |
| CSF2RA | -0.6529 | -2.0460 | 0.7401 | 3.463 | 3.565e-01 | 6.044e-01 |
| DHRSX | -0.8452 | -1.2770 | -0.4134 | 3.668 | 1.530e-04 | 3.112e-02 |
| GTPBP6 | -0.7816 | -1.0158 | -0.5474 | 4.286 | 4.001e-10 | 1.854e-06 |
| IL3RA | -0.6361 | -1.4900 | 0.2178 | 2.125 | 1.434e-01 | 3.903e-01 |
| IL9R | 0.7524 | -0.2612 | 1.7661 | -2.653 | 1.448e-01 | 3.919e-01 |
| P2RY8 | -0.8594 | -1.3393 | -0.3795 | 3.902 | 5.134e-04 | 6.524e-02 |
| PLCXD1 | -0.9169 | -1.6028 | -0.2311 | 2.952 | 9.040e-03 | 2.080e-01 |
| PPP2R3B | -0.9849 | -1.1792 | -0.7905 | 3.211 | 2.478e-19 | 3.886e-15 |
| SHOX | 1.3037 | -0.3854 | 2.9927 | -3.087 | 1.296e-01 | 3.719e-01 |
| SLC25A6 | -0.8070 | -1.1540 | -0.4599 | 6.582 | 7.984e-06 | 3.816e-03 |
| SPRY3 | -0.1323 | -0.4478 | 0.1833 | 2.435 | 4.095e-01 | 6.410e-01 |
| VAMP7 | 0.1490 | -0.1276 | 0.4256 | 4.233 | 2.894e-01 | 5.514e-01 |
| XG | -0.5035 | -1.4068 | 0.3998 | -0.464 | 2.730e-01 | 5.361e-01 |
| ZBED1 | -0.4506 | -0.6821 | -0.2191 | 5.722 | 1.667e-04 | 3.279e-02 |

logFC; log2 fold change, CI.L; confidence interval left, CI.R; confidence interval right, AveExpr; average expression, adj.P.Val;
